# Programmable Solid‐Electrolyte Interfaces for Efficient and Selective Electrochemical Hydrogenations

**DOI:** 10.1002/anie.2052091

**Published:** 2026-05-20

**Authors:** Anastasios Orestis Grammenos, Jessica Brandt, Yu Zhang, Zeen Wu, Mateusz M. Marzec, Sotirios Sotiropoulos, Piotr Jeleń, Jiayin Yuan, Markus Antonietti, Mateusz Odziomek

**Affiliations:** ^1^ Colloid Chemistry Department Max Planck Institute of Colloids and Interfaces Potsdam Germany; ^2^ Department of Chemistry Stockholm University Stockholm Sweden; ^3^ Academic Centre for Materials and Nanotechnology AGH University of Krakow Krakow Poland; ^4^ Department of Chemistry Aristotle University of Thessaloniki Thessaloniki Greece; ^5^ Faculty of Materials Science and Ceramics AGH University of Krakow Krakow Poland

**Keywords:** cation effect, electrochemical hydrogenation, electrochemical interface, electrode binder, poly(ionic liquid)

## Abstract

Electrode binders, typically regarded as passive mechanical additives, in fact define a solid‐liquid interface that governs charge screening and local proton‐electron transfer kinetics. Here we show that fluorine‐free poly(ionic liquid)s (PILs) operate as solid‐state electrolyte layers whose intrinsic electric fields modulate the competition between electrochemical hydrogenation (ECH) and the hydrogen evolution reaction (HER). When applied to Pd‐C catalysts, PIL binders reshape the electric double layer by repelling alkali cations and modulating interfacial pH, which change the kinetics of proton‐electron transfer, suppress Tafel hydrogen recombination and promotes selective coupling of adsorbed hydrogen with organic substrates. The resulting electrodes achieve up to fivefold higher ECH yields and fourfold greater Faradaic efficiencies than those based on Nafion or PVDF in three different pH values 0.6, 5.2, and 13, while simultaneously reducing Pd leaching. These findings identify the polymer additives more than a binder, but rather as an active field‐modulating medium. A solid analogue of the electrolyte double layer, thus extending classical electrolyte‐effect concepts to polymer‐confined interfaces and offering a strategy for binder‐controlled interfacial design in electrosynthetic systems.

## Graphical abstract

Poly(ionic liquid) binders are shown to actively control the electrode microenvironment, strongly enhancing electrochemical hydrogenation compared to traditional binders. The work redefines the binder as an active component of catalytic electrode design, reshaping local potential gradients and interfacial ion distribution to modulate adsorbed H coverage, suppress HER, and promote selective electrochemical hydrogenation.

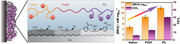

## 1 Introduction

Electrocatalytic transformations that rely on coupled proton‐electron transfer (PET) steps, such as the hydrogen evolution reaction (HER), carbon dioxide reduction (CO_2_RR), or organic electrohydrogenation (ECH), are governed by interfacial phenomena within the electric double layer (EDL) [1, 2, 3, 4, 5, 6, 7]. At this nanoscale boundary, local electric fields, ion distributions, and solvent structure collectively determine reaction energetics and selectivity [7, 8, 9, 10]. Recent advances have established that the electrolyte composition and the potential of zero charge (PZC) can tune these parameters, offering routes to control PET kinetics beyond conventional catalyst design [7, 10, 11]. However, while attention has focused on the liquid side of the interface, the solid phase, specifically the polymer binder that permeates the catalyst layer, remains an underexplored but equally decisive component of this electrostatic environment.

Binders are typically regarded as inert mechanical matrices that ensure adhesion and ionic transport within electrodes. Yet, occupying 5–20 wt.% of the catalyst layer, they necessarily participate in defining interfacial charge, wettability, and ion accessibility [12, 13, 14, 15, 16]. Classical fluorinated ionomers such as Nafion impose strong local electric fields through fixed sulfonate groups balanced by alkali cations, thereby reshaping interfacial pH and ionic composition [17, 18, 19]. These interfacial effects, often treated as secondary, closely mirror the mechanisms captured by the three prevailing frameworks describing electrolyte influences in electrocatalysis [20]: (i) modulation of adsorbate binding energies by noncovalent interactions, (ii) tuning of reaction barriers by surface electric fields and PZC, and (iii) solvent or ion reorganization within confined interfacial regions [1, 9, 10]. In these frameworks, polymer binders can be viewed as solid‐state extensions of the electrolyte, capable of exerting comparable control over interfacial structure and PET energetics.

Fluorine‐free poly(ionic liquid)s (PILs) represent a versatile platform for testing this concept. Their ion‐pair‐rich backbones and tunable cation chemistry combine strong adhesion with high ionic mobility [21, 22, 23, 24, 25, 26]. Their positive charge allows active participation in charge compensation at the cathode surface. By engineering the binder's electrostatics rather than its mechanical properties, one can deliberately shape local electric fields and transient pH, thereby influencing the reactions' selectivity. This notion parallels recent efforts to use external bias or electrolyte engineering to manipulate interfacial electric fields, [3, 4, 7, 8, 11] but translates those strategies into a solid, self‐assembled polymer layer intimately coupled to the catalyst surface.

Although binder‐mediated microenvironment effects on electrocatalytic interfaces have been reported previously, these studies have focused on commercial binders and small‐molecule electrocatalysis, including HER, ORR, CO_2_RR, and OER [18, 27, 28, 29]. In organic electrosynthesis, and particularly in electrochemical hydrogenations, the binder is not yet treated as a controlled interfacial variable, despite the greater mechanistic complexity arising from concurrent substrate adsorption, proton–electron transfer, hydrogen coverage, organic mass transport, and the competing HER. This creates an opportunity to control not only the activity, but also the selectivity between hydrogen evolution and hydrogen transfer to organic substrates.

Here, using commercial Pd‐C as a benchmark catalyst to isolate binder effects from catalyst‐design variables, we show that the chemistry of the binder, exemplified by poly(ionic liquid)s, programs the interfacial field and ion distribution on Pd‐C electrodes for electrochemical hydrogenations. The imidazolium‐based PIL suppresses HER by screening the electrode charge and repelling hydrated electrolyte cations, resulting in reduced proton activity and altered Volmer–Tafel kinetics. Simultaneously, the positively charged PIL layer restricts proton replenishment and retains acetate anions, leading to more pronounced and longer‐lived local alkalization during operation and increases the surface coverage of organic substrates, promoting selective hydrogenation of C═O, C═C, and C≡C bonds. The resulting Pd‐C/PIL electrodes deliver up to fivefold higher yields and fourfold greater Faradaic efficiencies than those using Nafion or PVDF at three different pH values (0.6; 5.2; 13.6) for three different reactions, while reducing Pd leaching. These findings define a broader paradigm: the polymer binder operates as a programmable solid‐state electrolyte that shapes the local electric‐field landscape and dictates proton–electron transfer kinetics. This concept bridges catalyst, electrolyte, and interface design, providing a practical route toward sustainable, fluorine‐free, and mechanistically tunable electrodes for selective electrosynthesis.

## 2 Results and Discussion

### 2.1 ECH of Benzaldehyde in Acetate Buffer

**FIGURE 1:**
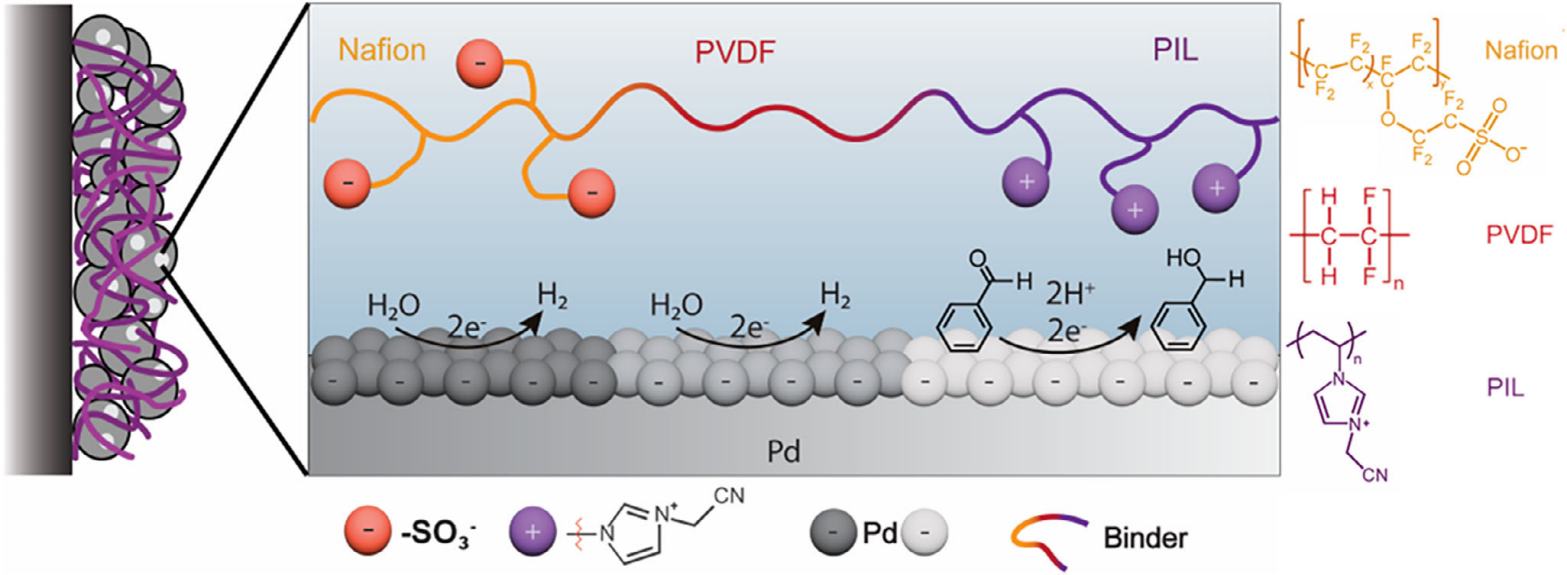
Programming the solid‐electrolyte interface through binder choice. Schematic representation of the HER/ECH selectivity on Pd‐C electrodes as governed by the microenvironment and the electric double‐layer (EDL) chemistry determined by the binder. Binders used in this study: Nafion (orange), PVDF (red), and PIL (purple).

Palladium nanoparticles (NPs) on carbon (Pd‐C) serve as a benchmark system for hydrogenation reactions [30], and their HER kinetics are well understood in terms of the Volmer‐Tafel mechanism [31, 32]. By embedding these NPs in polymer binders of contrasting electrostatic character, negatively charged Nafion, neutral PVDF, and positively charged imidazolium‐based PIL, we investigated how the solid electrolyte environment influences PET pathways (Figure 1). To that end, electrodes prepared with a PIL binder, specifically with PIL‐Im‐CN (poly(1‐vinyl‐3‐cyanomethylimidazolium bis(trifluoromethylsulfonyl)imide), having an imidazolium ring that bears a nitrile group attached via a methylene bridge (Figure S1), were compared against those with the benchmark commercial binders Nafion and PVDF. The synthesis and characterization of PILs has been reported elsewhere [33], and is also included in the experimental section, along with FTIR, Raman ^1^H NMR and thermogravimetric characterization in Figure S1. A schematic of the electrode architecture and electrochemical processes is shown in Figure 1.

**FIGURE 2:**
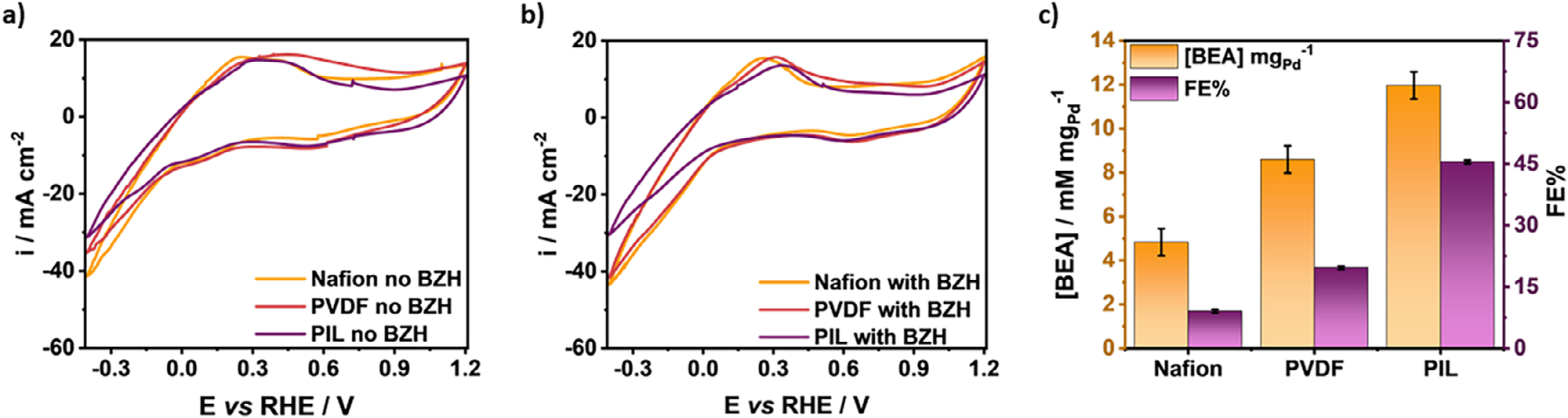
ECH of BZH with different binders. CVs of Pd‐C on GC using Nafion, PVDF or PIL binders in 3 M acetate buffer (pH 5.2) without (a) and with 20 mM of BZH (b), 50 mV s^−1^. c) Comparison of BEA yield and the respective FEs produced after 2 h of potentiostatic electrolysis at −0.1 V versus RHE without iR compensation, in 3 M acetate buffer with 20 mM BZH, using Pd‐C on carbon paper, with different binders.

The resulting electrodes were tested for the ECH of carbonyl groups [34, 35], with benzaldehyde (BZH) as the model substrate. Acetate buffer has been used as the electrolyte based on multiple previous works [30, 36, 37, 38], and also since it does not catalyze aldehyde side‐reactions (e.g. gem‐diol formation and/or the Cannizzaro reaction, Figures S2, S3). The preliminary cyclic voltammograms (CVs) presented in Figure 2 exhibit characteristic Pd voltammetric features for each binder, both without (Figure 2a) and with BZH (Figure 2b). All potentials within this manuscript are expressed versus RHE, unless stated differently. In the absence of BZH, a reduction shoulder appears at approximately +0.1 V attributed to the underpotential deposition (UPD) of hydrogen [36, 39, 40, 41]. Additionally, a broad oxidation peak related to Pd‐H oxidation is observed between +0.15 and +0.4 V. Upon addition of BZH (20 mM), the UPD hydrogen peak decreases due to competing BZH adsorption [36, 42, 43]. The CVs revealed progressively suppressed Pd‐H formation (and further re‐oxidation) as the binder charge shifted from negative to positive, suggesting that the local population of adsorbed H decreases in the presence of PIL.

The performance of Pd‐C/PIL electrodes was compared to their Nafion (Pd‐C/Nafion) and PVDF (Pd‐C/PVDF) counterparts by conducting 2‐h potentiostatic electrolyses for BZH ECH. Scanning electron microscopy (SEM) images of the Pd‐C/binder electrodes are shown in Figure S4, and the different binders did not change significantly the catalyst deposits on the carbon paper current collector. BZH and the hydrogenation product, benzyl alcohol (BEA), were detected and quantified via HPLC (Figure S5).

Control experiments without an applied potential or using bare carbon paper (the cathode current collector) resulted in no formation of BEA (Figures S6, S7). Preliminary findings indicated that −0.1 V was the optimal potential, yielding high product yields and satisfactory FE (Figure S8). FE and yield both increased at higher BZH concentrations, most likely due to higher coverage of active sites by the organic compound, increasing the rate of its surface reaction with adsorbed H while attenuating surface recombination of the latter as per the competing HER (Figure S9). For comparative studies, we have chosen the unoptimized 20 mM solution of BZH electrolyzed at −0.1 V, keeping the same Pd‐C/binder loading for each electrode (Table S1).

Among the three investigated binders, the current flowing during the electrolysis was the lowest in the case of Pd‐C/PIL (Figure S10), while yielding the highest amount of BEA (Figure 2c). The yields increased from 4.8 mM mg_Pd_^−1^ for Nafion and 8.6 mM mg_Pd_^−1^ for PVDF to 12 mM mg_Pd_^−1^ for PIL. An even larger difference was observed for FE, where the Pd‐C/PIL electrode exhibited a FE of 46 %, while Pd‐C/Nafion and Pd‐C/PVDF yielded a FE of 9% and 19%, respectively. This indicates that ECH is significantly more favored over HER when using the PIL binder. These experiments were performed conducting iR‐uncorrected electrolysis as it provides a more technologically relevant comparison, including the innate resistivity of different electrodes. To account for binder‐dependent variations in uncompensated resistance, we also performed electrolysis with iR compensation using the dynamic current‐interruption method. This procedure corrects intrinsic ohmic losses and, through periodic current interruptions, minimizes hydrogen‐bubble build‐up that can block active sites during gas‐evolving reactions [44]. Under these conditions, the yields for the Pd‐C electrodes were 13.5 mM mg_Pd_^−1^ for Nafion, 15.3 mM mg_Pd_^−1^ for PVDF and 20.1 mM mg_Pd_^−1^ for PIL. Additionally, the Pd‐C/PIL cathode maintained a FE of 41%, versus just 9% for Pd‐C/Nafion and 14% for Pd‐C/PVDF, speaking for a fourfold enhancement (Figure S11a). When iR drops were removed, the total electrolysis currents increased markedly for the Nafion and PVDF systems (Figures S10 and S11b), although the relative ratio of HER to ECH remained unchanged. Worth mentioning is that all electrodes shared similar uncompensated resistances (R_u_, Figure S12), indicating that bulk electrode resistivity does not explain the current‐density differences.

The choice of binder also had a marked impact on electrode stability. Dissolution of metal catalysts is a well‐known challenge in electrochemical systems [45]. To assess binder‐mediated mitigation of Pd leaching, we quantified dissolved Pd by ICP‐OES after two hours of electrolysis. The electrodes prepared with PIL and PVDF binders lost only 3.2% and 2.9% of their initial Pd loading, respectively, whereas Nafion‐bound electrodes lost 6.3% (Figure S13). This roughly 50% reduction in metal dissolution underscores the strong interaction between PILs and Pd nanoparticles, which enhances catalyst retention.

The Pd‐C/binder stability was assessed by a 24 h electrolysis (Figure S14a), for PIL and Nafion binders. A consistent declining performance was noted over time, indicating that the Pd catalyst is deactivated, as shown by its decreasing H_ads_ capacity but not its overall charge transfer properties (Figure S14b,c). Scanning electron microscopy (SEM) and energy dispersive x‐ray spectroscopy (EDX) did not reveal any significant changes on the electrodes’ morphology and composition before and after the stability tests (Figures S15–S18). Nonetheless, the increased Pd‐C/PIL binder performance was retained over that of Nafion (Figures S14d,e), along with its reduced Pd leaching (Figure S14f).

To determine whether chemical structure or net charge governs the ECH enhancement, we first compared PIL variants with different side‐groups, and counter‐ions. PILs featuring 1,2,4‐triazolium cations (instead of imidazolium) exhibited enhanced BEA yields, showing that tailoring the PIL's chemical structure leads to even higher yields. Conversely, substituting the nitrile with phenyl side groups led to a small decrease in ECH activity (see Figures S19 and S20). Likewise, swapping TFSI^−^ for Br^−^ only yielded a minor decrease in yield, showing that Br^−^‐based, fluorine‐free PILs perform similarly, and their FEs remained consistent, excluding the special effect of TFSI^−^ anions and driving the PIL binder away from fluorine‐containing polymers. Overall, every different PIL tested here performed significantly better than Nafion and PVDF, ruling out their specific functional moieties or anions as the primary driver of enhanced ECH. Thus, the PIL layer's positive charge was identified as the key factor enhancing the selectivity for electrochemical hydrogenation.

To test this hypothesis, we benchmarked Pd‐C/PIL against Pd‐C/Fumion, a commercial anion‐exchange ionomer bearing quaternary ammonium sites. Under both iR‐compensated and uncompensated electrolyses, the Pd‐C/PIL electrode delivered superior hydrogenation yields and FE (Figures S21a,b), despite showing slightly higher R_u_ (Figures S21 c,d). Pd‐C/Fumion's FE fell from 30% to 20% when ohmic losses were removed, reflecting an increased propensity for HER. In conclusion, Pd‐C/Fumion performance clearly lags behind that of Pd‐C/PIL, but show improved performances compared to Nafion and PVDF, especially in terms of FE, confirming that a positively charged binder layer is the main reason for suppressed HER and favored ECH. The difference between Fumion and PIL likely originates from the higher local charge density. Imidazolium and triazolium cations hold their positive charge in a more exposed and localized region of the molecule (around the ring), so the charge interacts more strongly with nearby anions, solvent molecules, or the catalyst surface. In contrast, quaternary ammonium cations in Fumion have their charge buried under bulky alkyl groups, which shield the positive center and weaken electrostatic interactions with the interface.

### 2.2 Controlling ECH Selectivity via Tuning the Kinetics of the Volmer‐Step and Pd‐H Coverage

**FIGURE 3:**
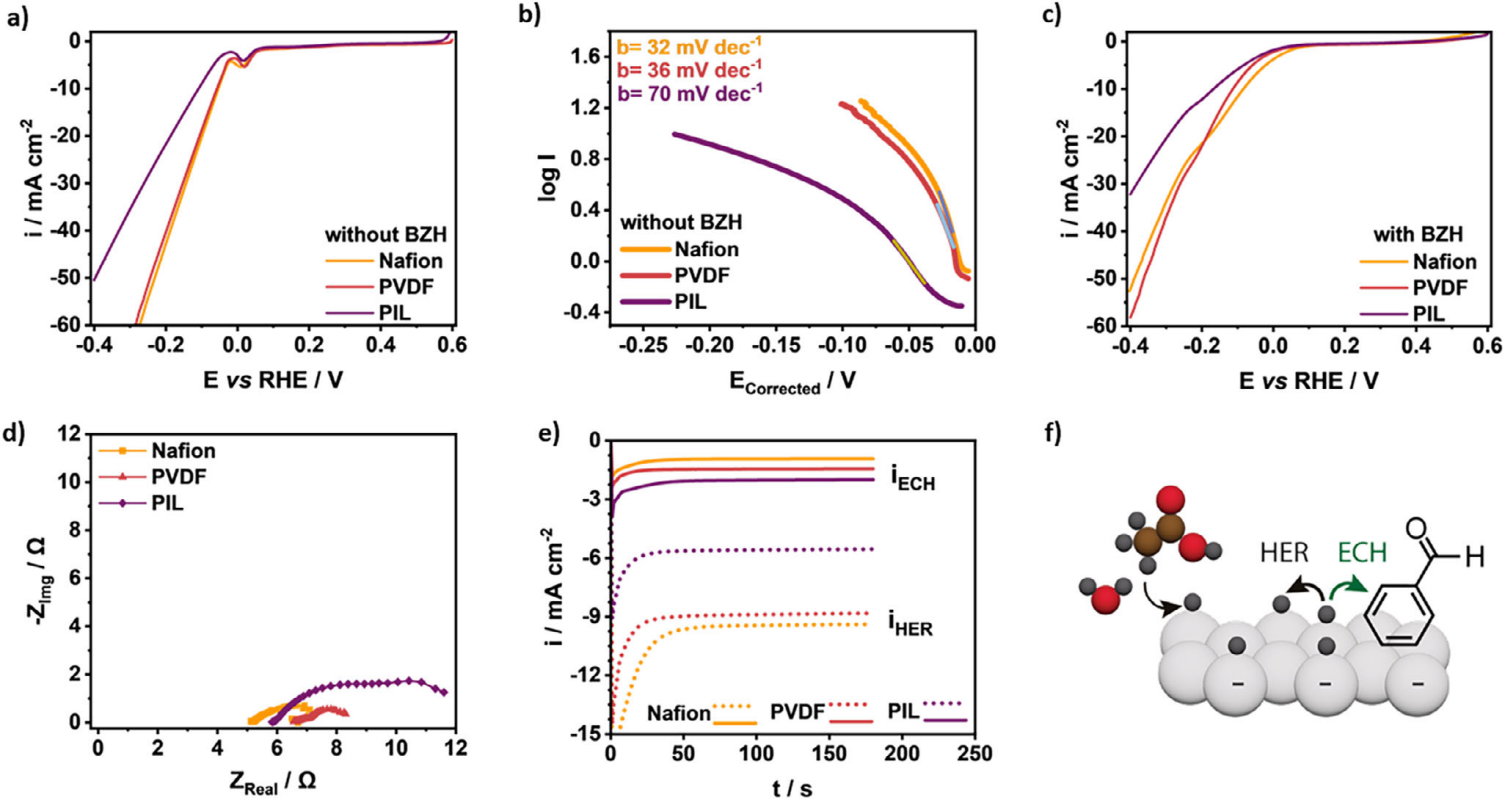
Electrochemical investigations using RDE. RDE LSVs of Pd‐C with Nafion, PVDF, PIL binders, 1600 rpm, 5 mV s^−1^ in 3 M acetate buffer (a) without BZH (b) corresponding iR‐corrected Tafel plots and slopes (c) LSVs with 20 mM BZH. (d) Nyquist plots at −0.1 V of Pd‐C/binder on carbon paper before BZH electrolysis. (e) Chronoamperometric curves for partial current densities i_HER_ (dotted) and i_ECH_ (solid) in the presence of 20 mM BZH, on a RDE GC disk, −0.1 V versus RHE, 1600 rpm. Partial current densities obtained by multiplying the current total current density (Figure S24) by the FEs in Figure 2c. (f) Schematic depiction of HER and ECH competition for H_ads_.

To elucidate the origin of the enhanced ECH efficiency of Pd‐C/PIL electrodes, we performed linear sweep voltammetry (LSV) using a rotating disk electrode (1600 rpm, 5 mV s^−1^) and compared the results with Pd‐C/Nafion and Pd‐C/PVDF analogues (Figure 3a). In the absence of BZH, all electrodes exhibited a Pd‐H formation feature near −0.05 V versus RHE, whose intensity decreased systematically as the binder charge shifted from negative to positive, indicating a lower surface population of adsorbed H in the presence of PIL. Kinetic analysis of iR‐corrected data (Figure 3b) yielded Tafel slopes of ∼30 mV dec^−1^ for Nafion and PVDF, consistent with a Volmer–Tafel pathway where the latter, hydrogen recombination, is rate‐determining [31, 32]. In contrast, the slope increased to 70 mV dec^−1^ for the PIL electrode, signaling mixed control by both the Volmer and Tafel steps [46]. The change of the electrochemical reaction kinetics for Pd‐C/PIL is thus related to the hindrance of the Volmer step with its rate becoming of the same order as that of the Tafel step.

Introducing 20 mM BZH eliminated the Pd‐H feature and produced a broad reduction shoulder extending up to −0.2 V (Figure 3c), characteristic of BZH ECH. Correspondingly, Tafel slopes increased to 164 mV dec^−1^ for Nafion and 222 mV dec^−1^ for PIL (Figure S22c), consistent with reduced electron‐transfer kinetics and/or site blocking by the adsorbed substrate. The mechanism of BZH ECH is well‐documented in the literature and further detailed in Supporting Information [30, 47, 48]. Overlapping RDE LSVs at different rotation rates (Figure S23) confirm that the reaction is under kinetic control over the entire potential range studied rather than mixed or mass transport control [49]. These observations indicate that the fundamental ECH mechanism remains binder‐independent, with differences in performance primarily originating from different kinetics of electrochemical step, thus the Volmer step. Electrochemical impedance spectroscopy (EIS, Figure 3d) corroborated this picture: at −0.1 V, Pd‐C/PIL displayed a markedly higher charge‐transfer resistance than its counterparts. This reflects the slower Volmer step (PET) (the only electrochemical step in both ECH and HER) and consequently lower overall HER currents.

Chronoamperometry at −0.1 V (no iR compensation, 1600 rpm, 3 M acetate buffer) (Figure S24) further confirms significantly lower HER currents for Pd‐C/PIL electrodes (‐8.6 mA cm^−2^) than for the other two binders (−20.1 and −20.7 mA cm^−2^ for Nafion and PVDF, respectively). Upon introducing BZH, total combined ECH and HER currents decreased to −5 mA cm^−^^2^ for Pd‐C/PIL and −11 mA cm^−^^2^ for Nafion and PVDF (Figure S24). Despite the lower overall currents, the partial current for ECH is the highest for Pd‐C/PIL (Figure 3e) confirming that the binder surpresses parasitic HER and enhances the reaction of interest.

ECH proceeds through sequential adsorption and surface combination steps, while HER is dominated by the Volmer–Tafel pathway. The kinetics of the Volmer step govern both processes, as they compete for the same surface‐bound hydrogen species, H_ads_ (Figure 3f). Because both reactions share the Volmer step in their mechanisms (see Supporting Information) and compete for the same active sites, the relative rates of HER and ECH depend primarily on the fractional surface coverages of hydrogen and benzaldehyde, as expressed in Equation 1a,b.

(1a,b) $$\;r_{\mathrm{HER}}=k_{\mathrm{HER}}\;\theta_{H}^{2}\;\;r_{\mathrm{ECH}}=\;k_{\mathrm{ECH}}\theta_{H}\theta_{\mathrm{BZH}}$$

Where: *θ_BZH_*—fractional surface coverage by BZH, *θ_H_*—fractional surface coverage by H_ads_, *k_HER_*—rate constant of HER, *k_ECH_*—rate constant of ECH, *r_HER_*—reaction rate of HER, *r_ECH_*—reaction rate of ECH

Assuming that the surface is shared between adsorbed hydrogen and substrate (*θ_BZH_* + *θ_H_* = 1), an increase in *θ_BZH_* enhances the rate of ECH, while simultaneously suppressing hydrogen evolution. Both reactions originate from the same PET (Volmer) step, but the distinct Tafel slopes observed for HER, ∼30 mV dec^−1^ for Nafion/PVDF versus 76 mV dec^−1^ for PIL, indicate that the apparent rate‐determining step varies with the interfacial environment imposed by the binder. The increase in Tafel slope can be interpreted as reduced kinetics of PET (Volmer step) and thus reduced *θ_H_* ​. We can assume that, in the presence of BZH, the trend remains similar, i.e., the kinetics of PET are slower for Pd‐C/PIL than for the other samples. This is also indicated by the higher Tafel slopes measured in the presence of BZH; however, the values are too high for an unequivocal mechanistic interpretation. A reduced *θ_H_* shifts the balance toward substrate hydrogenation, leading to the experimentally observed trend r_ECH_ (Nafion<PVDF<PIL). The higher ECH selectivity of Pd‐C/PIL thus reflects diminished hydrogen coverage and enhanced BZH adsorption at the positively charged, cation‐excluding interface. Alternatively, increased *θ_BZH_* due to the higher BZH concentration also increased both FE and yield of ECH (Figure S9).

Concluding, the suppressed HER activity of Pd‐C/PIL cannot be explained solely by competitive adsorption with BZH. Even in its absence, the electrode exhibits slower PET kinetics, as reflected by a larger Tafel slope and higher charge‐transfer resistance in EIS. This indicates that the positively charged PIL intrinsically hampers the Volmer step. Since XPS analysis revealed no significant changes in the electronic properties of Pd (Figure S25), these differences must arise from variations in the local electrolyte composition and acid‐base equilibria rather than from intrinsic catalyst modification. Contact angle measurements were conducted on the different Pd‐C/binder electrodes. The contact angle decreased in the order Nafion> PVDF> PIL, with water drops directly permeating the Pd‐C/PIL electrode (Figure S26). We expect that hydrophilicity differences do not crucially affect HER in this case, as indicated by the electrodes’ similar voltammograms. Moreover, the increased hydrophobicity should decrease Nafion's and PVDF's HER performance [50]. Thus, hydrophobicity is ruled out as a factor of the observed HER suppression, which should stem from chemical and electrostatic changes at the electrode–electrolyte interface and not from lower water concentration. To probe these microenvironmental effects directly, we next examined transient pH variations using potentiometric measurements, linking binder‐induced local pH changes to the observed modulation of HER and ECH kinetics.

### 2.3 The Impact of Binders on the Transient pH

**FIGURE 4:**
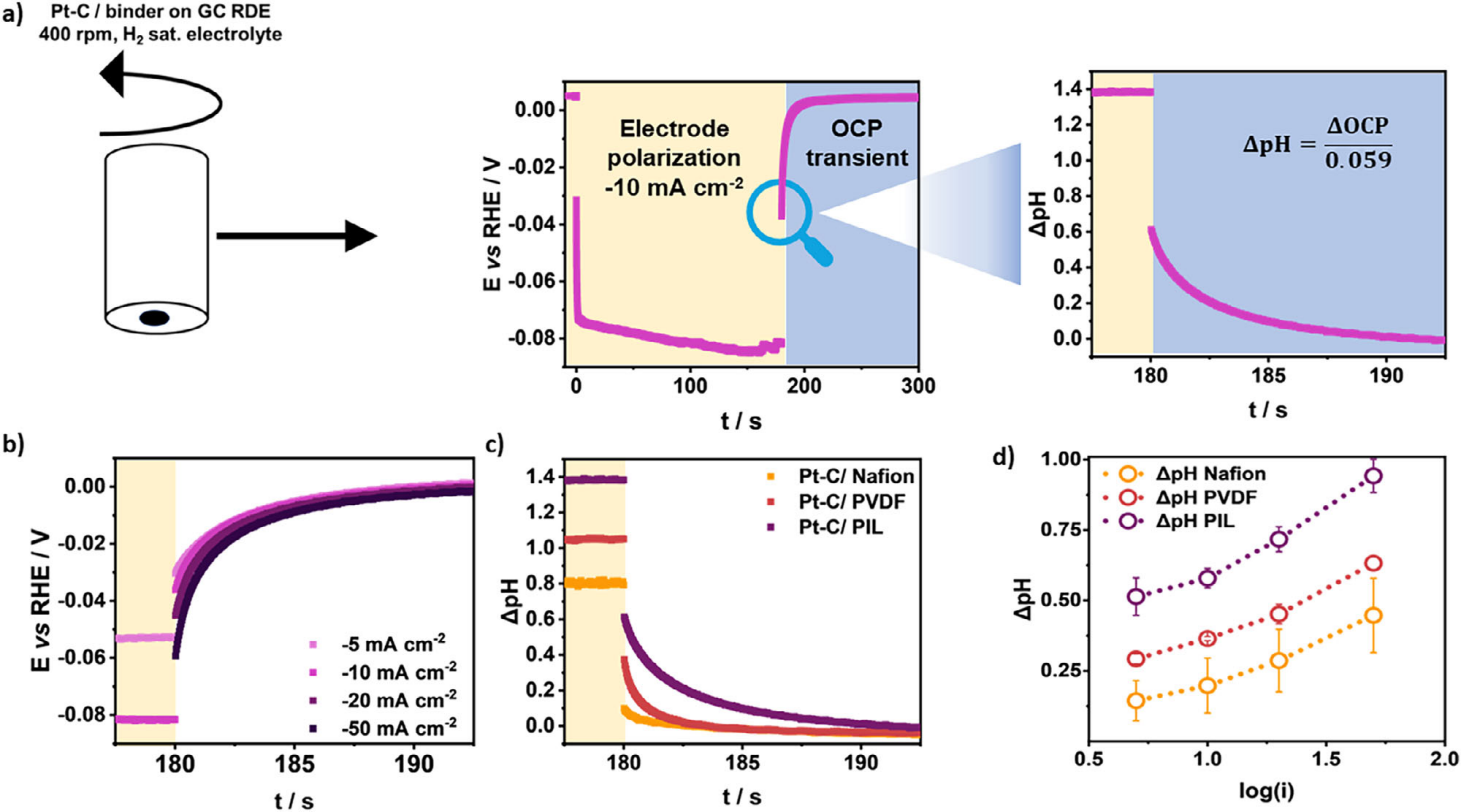
pH swing experiments on Pt‐C/binder. a) transient pH measurement procedure. b) OCP decay on Pt‐C/PIL for different current densities. c) changes in pH for Pt‐C/binder electrodes, after polarization at −10 mA cm^−2^. d) changes in pH for Pt‐C/binders after polarization at −5, −10, −20 and −50 mA cm^−2^, ΔpH plotted against the logarithm of current densities. All measurements conducted in H_2_ saturated 3 M acetate buffer, 400 rpm, with the Pt‐C/binder catalysts drop casted on a GC RDE electrode. Yellow‐shaded parts in graphs a‐c indicate electrode polarization.

The changes in interfacial pH during electrolysis can have a dramatic impact on the kinetics of the Volmer step [51]. We monitored local pH variations using a recently reported method by Sauve et al. [17], for an RDE setup (Figure 4a). Given the rapid kinetics of HER and the reverse hydrogen oxidation reaction (HOR) on Pt, and its stable electrochemical potential, it serves as a pH sensor under H_2_ atmosphere, based on the Nernst equation. Therefore, local pH swings can be quantified by recording transient potentials at open‐circuit conditions immediately following chronopotentiometry (Equation 2, for more details see methods in SI). As Pt offers fast kinetics of HER/HOR, we performed experiments using Pt NPs on the same carbon support as Pd, deposited on a GC RDE using the same investigated binders. HER was conducted at different current densities in H_2_‐saturated 3 M acetate buffer.

(2) $$\Delta pH\;=\frac{\Delta OCP}{0.059}$$

Figure 4a shows an open‐circuit potential (OCP) decay (blue shaded part) for Pt‐C/PIL observed directly after polarization (yellow shaded part). The shift of the OCP to more negative values directly after polarization reflects the shift of the redox potential of HER/HOR pair due to the local pH increase. At higher current densities, HER occurs at higher rates, forcing a greater pH increase, ranging from 0.51 to 0.94 for −5 and −50 mA cm^−2^ respectively, as reflected by the larger OCP transients (Figure 4b). The local alkalization was observed for all 3 binders (Figure S28), with varying extents of pH change. After evolving H_2_ at −10 mA cm^−2^, the detected changes in pH followed the order PIL> PVDF> Nafion, with their respective ΔpH values being 0.58, 0.37, and 0.20 (Figure 4c). The pH changes were less than one unit in all instances, given the concentrated buffer (3 M) employed as an electrolyte. Buffer solutions minimize pH swings, as also demonstrated previously, with 1 M phosphate buffers resulting in ΔpH < 2 [17], making the suppression of pH changes below 1 plausible in the case of the employed 3 M acetate buffer. This shows that whenPIL is employed as the electrode binder, the increase of pH at the Pt/C catalyst interface is indeed higher. Furthermore, the equilibration of OCP transients for PIL takes longer time than other two binders, with clearly extended sloping region. The difference in transient pH remained consistent across all current densities, confirming that it stems from the imposed changes to the electrodes’ local environment by the employed binder (Figure 4d, S28). Furthermore, the experiments were conducted in triplicate for each binder type and showed that the aforementioned differences are persistent (Figure S29). Since the rate of reactions was set even for three binders the difference in transient pH comes from the difference in diffusion/permeation of ions/molecules through the binder.

Given the high buffer concentration, and the corresponding high acetic acid concentration (∼1 M), the Volmer step of HER evolution proceeds either through reduction of H^+^ from acetic acid dissociation or through direct acetic acid reduction. Worth mentioning is that in the immediate vicinity of the electrode, the concentration of H^+^ is very limited (6.3*10^−6^ M), thus it is quickly consumed and has to be either replenished by dissociation of acetic acid or by changing the proton source directly to acetic acid. In fact, it has been already discussed that the thermodynamics of both processes are identical [52, 53]. Water reduction can occur to a lower extent as acetic acid is a much better proton source with pK_a_ of 4.76 compared to 14.0 for water. Therefore, the main mechanism of changing pH at the electrode surface corresponds to the change of acetate ion to acetic acid ratio, according to Henderson–Hasselbalch equation describing the pH of buffered solutions (Equation 3).

(3) $$pH=pK_{a}+log_{10}\;\left(\frac{\left[A^{-}\right]}{\left[\mathrm{HA}\right]}\right)$$

This tells us that the effective concentration of acetate species is higher at the interface of the Pt‐C/PIL electrode during operation, which hampers the dissociation of acetic acid, requiring higher overpotentials to reach the same current density as in Pt‐C/Nafion and Pt‐C/PVDF. This might be associated with the positively charged imidazolium cations retaining acetate anions within the film/hindering proton replenishment at the electrode vicinity. The higher acetate‐to‐acetic acid ratio observed with the PIL binder under electrochemical polarization was also experimentally confirmed by in situ Raman spectroscopy (Figure S30). The qualitative mathematical expression of the pH impact on the Volmer step can be found in Equations S1–S3. Importantly, the use of model HER catalysts, specifically Pt NPs with different binders confirmed the trends initially observed with Pd NPs. At the same current densities, Pt‐C/PIL showed the highest overpotentials, clearly indicating that the PIL binder hampers HER kinetics, independently of the catalysts (Figure S31).

### 2.4 Sodium Versus Imidazolium Cations at the Interface

**FIGURE 5:**
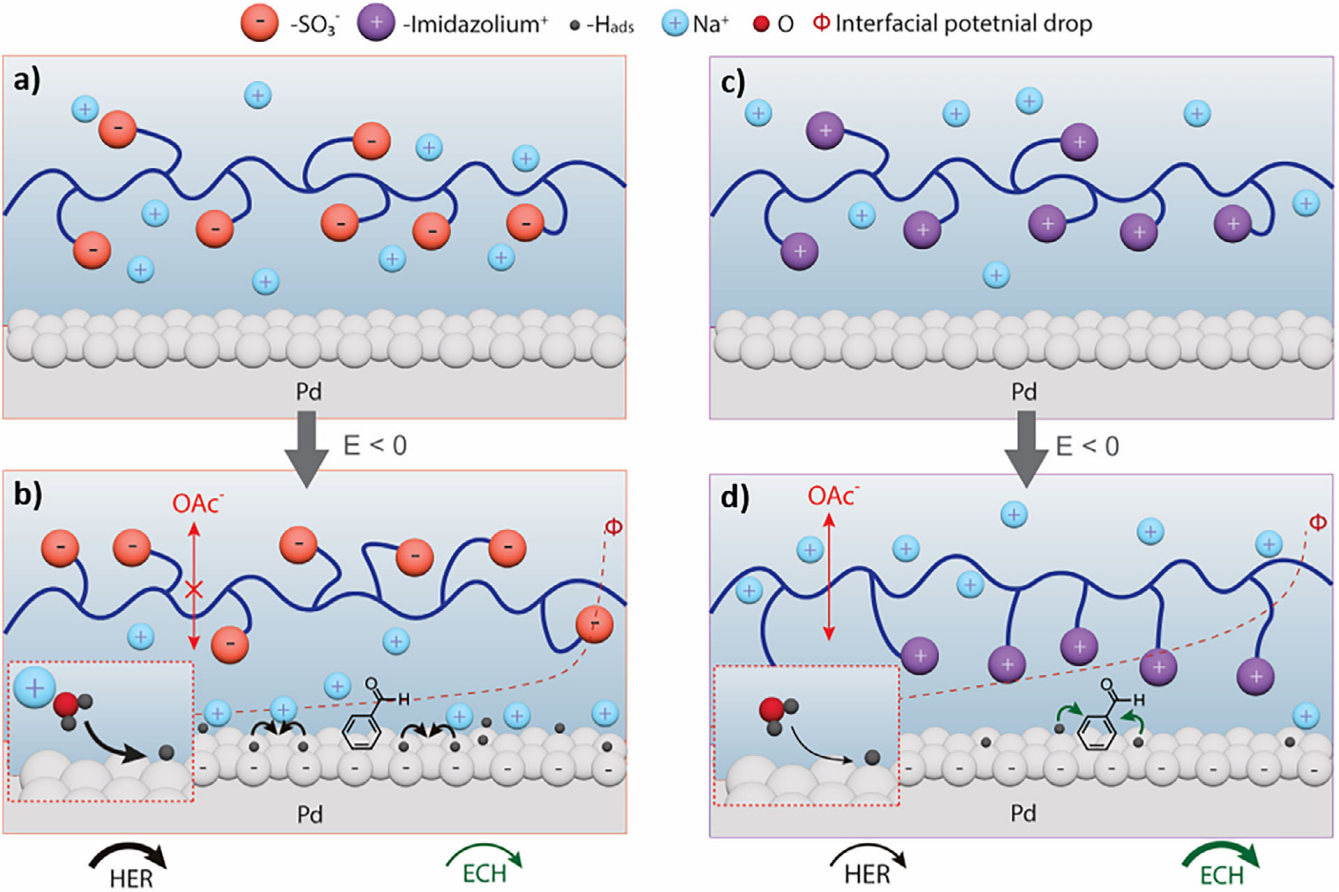
Schematic illustration of the Pd‐C/binder electrode‐electrolyte interface during the ECH of BZH. (a, b) Pd‐C/Nafion before and during cathodic polarization: the negatively charged electrode repels sulfonic anions, attracts Na^+^ ions (which readily permeate the negatively charged layer), and restricts acetate transport. The accumulated Na^+^ ions create steep potential gradients (ϕ) that facilitate the proton‐discharge Volmer step (inset b), increasing θ_H_ and thus promoting HER. (c, d) Pd‐C/PIL before and during cathodic polarization: the negatively polarized electrode attracts imidazolium cations, repels Na^+^ ions, and allows easier acetate transport. The imidazolium cations screen the surface charge over a longer distance, resulting in shallower potential gradients and less efficient proton discharge (inset c), which decreases θ_H_ and favors ECH.

The variations in HER and ECH kinetics originate from the interplay between the binder's charge and molecular architecture, which together define the local electrochemical environment under polarization. By modulating ion mobility and interfacial potential gradients, the binder restructures the electrochemical double layer, thereby governing the rates of PET. These processes are schematically described in Figure 5. Negatively charged Nafion attracts counter‐cations (e.g. Na^+^) that accumulate at the cathode, steepening electrostatic gradients and accelerating charge transfer, consistent with its lower charge‐transfer resistance in EIS (Figure 3d, S8). The neutral PVDF binder displays similar behavior due to its unimpeded ionic permeability. In contrast, the PIL's positively charged imidazolium units (partially) compensate the electrode potential and repel hydrated cations through Donnan exclusion [17, 54]. The resulting reduction in metal cation flux weakens the local electric field (potential gradient at the interface), increases charge‐transfer resistance, and slows the proton discharge. This intrinsic kinetic suppression explains the higher Tafel slope observed for PIL (∼70 mV dec^−1^) relative to Nafion and PVDF (∼30 mV dec^−1^).

Beyond electrostatics, chemical interactions also contribute. Elevated Na^+^ concentration on Pd surfaces is known to shorten Pd⋯H─O─H distances and facilitate the Volmer step [55], whereas cation exclusion within the PIL layer removes this promoting effect. The resulting lower hydrogen coverage suppresses H_ads_‐H_ads_ recombination, increasing chances for coupling between residual H_ads_ and adsorbed BZH, enhancing ECH selectivity. In other words, the surface concentration of H atoms is low for efficient HER but high enough for ECH. Reduced Na^+^ accumulation also prevents salting‐out of the organic substrate [56], maintaining its local concentration near the active surface. Simultaneously, the PIL matrix alters acid‐base equilibria by retaining acetate anions and hindering proton replenishment, creating a slightly more alkaline interfacial environment that further disfavors HER [51].

In situ Raman spectra (Figure S30) indicated changes in the shoulders at 900 and 930 cm^−1^, corresponding to acetic acid and acetate species, respectively. As can be seen in the case of PIL, the acetate/acetic acid ratio increases with increasing polarization compared to Nafion, indicating the changes in local ion concentration. The enhanced acetate concentration also aligns experimentally with the higher local pH values obtained for Pd‐C/PIL. Although Na^+^ accumulation is not probed directly from these measurements, the Raman spectra show that binders actively alter the local ion concentration. Together, these synergistic effects such as cation exclusion, charge screening, and micro‐pH modulation govern PET and indirectly the competition between HER and ECH. The resulting enhancement in FE underlines the binder's dual electrostatic and chemical role in shaping the reaction microenvironment.

Viewed through the framework of the three conceptual schools describing electrolyte effects in electrocatalysis, the binder can be regarded as their solid‐state analogue. Its positively charged matrix alters ion and adsorbate interactions (first school), modulates the local electric field, (second school), and constrains solvent orientation and dynamics within the confined interface (third school) [20]. Each of these effects is intimately linked to the cations that screen the interfacial charge and reshape the free‐energy landscape of PET, and thereby control the competition between HER and ECH. Yet, discriminating which contribution, field modulation, ion pairing, or solvent structuring, dominates under operating conditions remains unresolved, calling for systematic operando and theoretical studies to unravel their relative importance. Taken together, these insights reveal that binder chemistry operates as a solid‐state analogue of electrolyte engineering, where molecular design dictates interfacial electric fields, ion distributions, and proton activity. Thus, the binder emerges as a programmable solid‐electrolyte interface bridging catalyst, electrolyte, and interfacial design.

### 2.5 Expanding the Scope of Enhanced ECH Performance

**FIGURE 6:**
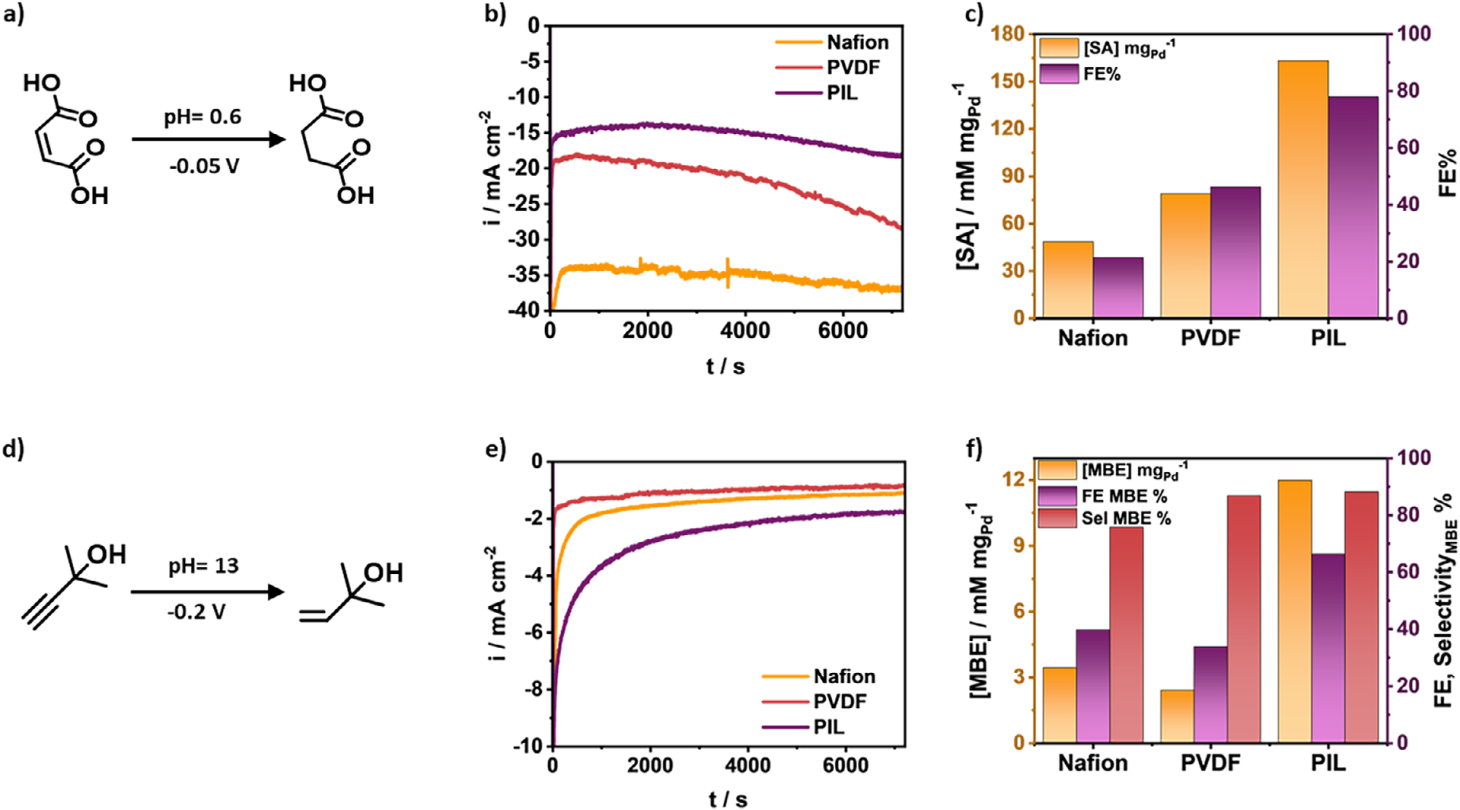
Expanding the scope of enhance performance with Pd‐C/PIL at various pH. a) Reaction schematic of MA ECH to SA. b) Chronoamperometric curves of Pd‐C/binder in 0.2 M H_2_SO_4_ during the potentiostatic electrolysis of 0.1 M MA at −0.05 V versus RHE. c) Comparison of yield and FE of SA production, after 2 h of MA bulk electrolysis at −0.05 V versus RHE for Pd‐C/binder electrodes. d) Reaction schematic of MBY (semi‐) ECH. e) Chronoamperometric curves of Pd‐C/binder in 0.1 M NaOH, during the potentiostatic electrolysis of 0.1 M MBY, at −0.2 V versus RHE. f) Comparison of yield and FE of MBE, and MBE selectivity after 2 h of bulk electrolysis on Pd‐C/binder at −0.2 V versus RHE in 0.1 M NaOH. All electrolyses were conducted using iR compensation through the dynamic current interrupt technique.

To assess the universality of the PIL binders’ enhanced ECH performance, we further expanded our study to different functional groups and pH values. Each bulk electrolysis was conducted using iR compensation through the dynamic current interrupt technique to compare the intrinsic activity of catalysts with different binders. At first, we chose the conversion of maleic acid (MA) to succinic acid (SA) as a technologically relevant [57, 58] model reaction, for the hydrogenation of C═C bond in acidic conditions (Figure 6a) [59]. Preliminary voltammetric investigations deemed the ECH of MA feasible, as shown in Figure S32. More specifically, the onset potential of MA hydrogenation was found to be 0.05 V.

The bulk electrolysis was conducted at −0.05 V, in 0.2 M H_2_SO_4_. The strongly acidic electrolyte (at approximately pH = 0.6) was employed to ensure that MA was in its undissociated state (pKa_1_ = 1.96), as described in our previous work [59]. The recorded currents were again significantly smaller for PIL binder (ca. −15 mA cm^−2^) compared to PVDF and Nafion, ca. −20 and −35 mA cm^−2^ respectively (Figure 6b). ^1^H NMR spectroscopy confirmed the exclusive formation of SA and enabled its quantification. As seen in Figure 6c, when using PILs as a binder, the SA yield was improved, compared to the Nafion & PVDF binders. Pd‐C/PILs reached 163.2 mM mg_Pd_^−1^ of product in comparison to 48.5 and 78.9 mM mg_Pd_^−1^ for Nafion and PVDF, under the same electrolysis conditions. Moreover, the Pd‐C/PILs electrocatalyst's FE was significantly enhanced, reaching near 80% in comparison to 45% for PVDF and 23% for Nafion, thus following the same trend as BZH ECH. This difference in SA yield and FE can be attributed to the electrostatic repulsion of H_3_O^+^ with the positively charged PIL, which limits the local availability of protons, thus hindering the competing HER.

At last, the performance of the sample with PIL binders in basic media was evaluated by the electrochemical semi‐hydrogenation reaction of 2‐methyl‐3‐butyn‐2ol (MBY) (Figure 6d). The C≡C bond of MBY can either hydrogenate partially, yielding 2‐methyl‐3‐buten‐2‐ol (MBE), or fully to 2‐methyl‐3‐butan‐2‐ol (MBA). The reaction has been shown to work most effectively in alkaline media [59, 60], so the employed electrolyte was 0.1 M NaOH. As can be seen from the voltammograms recorded, HER is significantly suppressed in alkaline media, compared to mildly or strongly acidic conditions (Figure S33). When MBY was added to the solution, an increase in reduction current was observed from −0.1 V onwards, thus confirming the MBY (*semi*‐)ECH feasibility.

The MBY ECH was conducted at −0.2 V and the products were detected and quantified through ^1^H NMR. MBE was confirmed to be the main product, with a fairly consistent selectivity across binders, ranging from 75% for Nafion to 85% for PVDF and PIL (Figure 6f). Strikingly, the yield of MBE produced was significantly higher in the case of Pd‐C/PIL (12 mM mg_Pd_^−1^) than for Nafion and PVDF (3.4 and 2.4 mM mg_Pd_^−1^, respectively), marking a fivefold increase compared to PVDF. At the same time an almost two‐fold increase in FE, from 35 % to 65 % in favor of Pd‐C/PIL was observed. Contrary to previous examples, Pd‐C/PIL yielded the highest currents in basic media (−2.6 mA cm^−2^ for PILs vs. −1.5 and −1.1 for Nafion and PVDF, respectively, Figure 6e).

We speculate that this intense increase in MBE production stems from the reduced local concentration of Na^+^ at the vicinity of Pd‐C/PIL, compared to Pd‐C/Nafion or Pd‐C/PVDF, as discussed above. Additionally, OH^−^ accumulation, favored by the positively charged PIL, could help the semihydrogenation, as shown elsewhere, where the reaction rate increases with increasing NaOH concentration in a certain range [60]. Nevertheless, additional studies need to be performed to understand this effect. Overall, the ECH of C═C and C≡C bonds in strongly acidic and alkaline environments, respectively, verifies that PILs consistently deliver an enhanced ECH performance, for different unsaturated bonds and different chemical environments.

## 3 Conclusion

This study demonstrates that the polymer binder is not an inert additive but an integral component of the electrochemical interface. By replacing conventional fluorinated ionomers with fluorine‐free PILs, we show that the binder forms a solid‐electrolyte interface whose electrostatic and chemical characteristics govern local PET kinetics. Binders with negative, neutral, and positive charge (Nafion PVDF and PIL, respectively) have a distinct impact on ECH with the positive charge leading to enhanced performance. This was consistent for different PILs (and Fumion), all surpassing the other binders. Moreover, we show that not only the binders’ charge, but also their chemical structure influences activity, depending on steric hinderance and the charged functional groups’ accessibility. The positively charged PIL matrix screens the electrode potential, repels alkali cations, and retains anions, thereby modulating charge distribution, interfacial pH, and solvent organization. These effects collectively suppress the Volmer step of hydrogen evolution and promote the selective hydrogenation of organic substrates, yielding higher FE and improved catalyst stability without altering the metal phase.

The results reveal that control traditionally achieved through electrolyte composition can instead be embedded within the solid electrode architecture itself. The polymer thus operates as a programmable solid‐electrolyte layer that bridges catalyst, binder, and electrolyte, defining the local reaction microenvironment and dictating the competition between HER and ECH. This concept extends classical electrolyte effect frameworks to polymer‐confined interfaces and provides a strategy for binder‐controlled interfacial design in electrochemical hydrogenations and related electrosynthetic systems.

Future developments may exploit chemically tailored ionic polymers to tune charge screening, ion pairing, and solvent orientation in other electrocatalytic systems. Integrating such programmable solid‐electrolyte interfaces with operando characterization and theoretical modeling could enable predictive control of interfacial reactivity and selectivity across a broad class of electrochemical transformations.

## Author Contributions

**Anastasios Orestis Grammenos**: investigation, data curation, writing – original draft, methodology, validation, formal analysis. **Jessica Brandt**: investigation. **Yu Zhang**: investigation. **Zeen Wu**: investigation. **Mateusz M. Marzec**: methodology. **Sotirios Sotiropoulos**: methodology. **Piotr Jeleń**: methodology. **Jiayin Yuan**: methodology. **Markus Antonietti**: writing – review and editing, resources, funding acquisition. **Mateusz Odziomek**: conceptualization, investigation, funding acquisition, writing – review and editing, writing – original draft, methodology, supervision, project administration.

## Conflicts of Interest

The authors declare no conflicts of interest.

## Supporting information




**Supporting File**: Electronic Supporting Information contains: Detailed experimental procedures; product‑quantification chromatograms; expanded electrochemical characterization (CV, LSV, RDE, and EIS) of all electrodes; additional electrolysis data; XPS spectra and analysis; in situ Raman experiments and a supplementary note on the general mechanism of benzaldehyde electrochemical hydrogenation on Pd in acetate buffer.

## Data Availability

The data that support the findings of this study are available from the corresponding author upon reasonable request.
