# Supplementary material for: Programmable Solid‐Electrolyte Interfaces for Efficient and Selective Electrochemical Hydrogenations
Source: Angew Chem Int Ed Engl. 2026 May 20;65(29):e2052091. doi: 10.1002/anie.2052091 (PMC13360868; doi:10.1002/anie.2052091)
Supplement: Supplementary file 1 — Supporting File: Electronic Supporting Information contains: Detailed experimental procedures; product‑quantification chromatograms; expanded electrochemical characterization (CV, LSV, RDE, and EIS) of all electrodes; additional electrolysis data; XPS spectra and analysis; in situ Raman experiments and a supplementary note on the general mechanism of benzaldehyde electrochemical hydrogenation on Pd in acetate buffer. [file ANIE-65-e2052091-s001.docx]

**Programmable solid-electrolyte interfaces for efficient and selective electrochemical hydrogenations**

Anastasios Orestis Grammenos,^1^ Jessica Brandt,^1^ Yu Zhang,^2^ Zeen Wu,^2^ Mateusz M. Marzec,^3^ Sotirios Sotiropoulos,^4^ Piotr Jeleń,^5^ Jiayin Yuan,^2^ Markus Antonietti,^1^ Mateusz Odziomek ^1,5*^

^1^ Colloid Chemistry Department, Max Planck Institute of Colloids and Interfaces, 14476 Potsdam, Germany

^2^ Department of Chemistry, Stockholm University, Stockholm, SE-10691, Sweden

^3^Academic Centre for Materials and Nanotechnology, AGH University of Krakow, A. Mickiewicza Ave. 30 30-059 Krakow, Poland

^4^ Department of Chemistry, Aristotle University of Thessaloniki, 54124, Greece

^5^ AGH University of Krakow, Faculty of Materials Science and Ceramics, al. Mickiewicza 30, 30-059 Krakow, Poland

E-mail: mateusz.odziomek@agh.edu.pl

**Keywords:** poly(ionic liquid), electrode binder, electrochemical hydrogenation, electrochemical interface, cation effect

1. **Experimental section**
   1. Materials **& Reagents**

1-Vinylimidazole (99%), bis(trifluoromethanesulfonyl)imide lithium salt (LiTf_2_N, 99.95%), 2,2’-azobis(2-methylpropionitrile) (AIBN, 98%), and 4-bromobutyronitrile (99%) were obtained from Sigma-Aldrich and used without further purification. Dimethyl sulfoxide (DMSO), acetonitrile (MeCN), ethylene glycol (EG), ethanol, acetone, tetrahydrofuran (THF), isopropanol, and deionized water were of analytic grade. The synthetic details and the structural characterization of the PIL can be referred to our previous report,^1^ and in short in experimental section 1.2.

Palladium on carbon powder (10 wt %), poly(vinylidene fluoride) (PVDF) (average M.W. ~534,000), and 2-methyl-3-butyn-2-ol (MBY, >98%) were purchased from Sigma-Aldrich. Maleic acid (MA, synthesis grade), benzaldehyde (BZH, synthesis grade) and sodium acetate buffer (3 M, pH 5.2) were purchased from Merck. Sulfuric acid (≥ 95%). Dimethylformamide (DMF) (>99%), and sodium hydroxide (analytical grade) were purchased from Thermo Fisher. Acetonitrile was purchased from Honeywell (LC-MS-CHROMASOLV grade, ≥ 99.9%) and Merck (LC-MS LiChrosolv grade). Nafion dispersion (Nafion D521 Dispersion, 5 *wt.%*) was obtained from Ion Power GmbH. Milli-Q water was used for the mobile phase for the HPLC quantifications. D_2_O (99.90%) was purchased from Eurisotop. Fumion FAA-3-SOLUT-10 dispersion (10 *wt.%*) was purchased from the Fuelcell store.

- 1. Poly(ionic liquid) PIL-Im-CN synthesis

The imidazolium-based PIL-Im-CN with a cyanomethyl substitute and a TFSI anion was prepared via polymerization of the ionic liquid monomer 1-vinyl-3-cyanomethylimidazolium bromide, followed by an anion exchange reaction with TFSI anion, similar to our previous method.^2^ In a typical polymerization procedure, 10 g of 1-vinyl-3-cyanomethylimidazolium bromide and 200 mg of AIBN were dissolved in 100 ml of DMSO in a round bottle flask and deoxygenated by three cycles of freeze-pump-thaw process. After backfilling with nitrogen, the reaction flask was kept at 90°C for 20 h. After cooling down to room temperature, the viscous reaction liquid was dropped into 1.5L of THF. The white precipitate was collected and re-dissolved in methanol before it was precipitated again in THF. The white powder was dried at 90 °C under vacuum (10-2 mbar) overnight. Its chemical structure was verified by its 1H-NMR spectrum shown in Figure S1. Next, this white powder was subject to a typical anion exchange process to replace Br^-^ by Tf2N^-^. The above-obtained white powder was dissolved in an aqueous solution by gentle heating at a concentration of 10 g/L. An aqueous LiTf2N solution at 50 g/L was dropped into the afore-mentioned aqueous solution under stirring, and a white precipitate was observed. The addition was continued till a final [Tf2N]/[Br] molar ratio of 1.15 was reached in the reaction mixture. The stirring continued for another 30 min. The white precipitate, namely the PIL-Im-CN with the TFSI anion, was collected by filtration, washed repeatedly with deionized water and dried at 90 °C under vacuum (10-2 mbar) overnight. The apparent molecular weight and polydispersity index of the PIL polymer was 1.15 × 105 g /mol and 2.95, respectively.

FTIR measurements were performed using Bruker Vertex 70v vacuum spectrometer in transmission mode (standard KBr pellet method). Spectral resolution was set to 2 cm^-1^ and 512 scans were accumulated. Raman measurements were performer using WITec Alpha 300M+ spectrometer equipped with 488 nm laser. Power was adjusted to 1 mW to prevent sample degradation and to suppress fluorescence. 600 grating was used. The measurement consisted of 1000 accumulations of 1 second scans. TGA was performed on a NETZSCHTG 209 F1 device in the range of 25°C to 900°C in a He atmosphere with a heating rate of 2 °C/min in a Pt crucible

- 1. Electrode preparation

For the voltammetric studies, Glassy Carbon (GC) Rotating Disk Electrodes (RDE) from Pine (5 mm diameter, surface area of 0.196 cm^2^) were polished with alumina powder slurries of 0.05 µm before the deposition of the electroactive materials. 3.40-3.45 mg of ground Pd-C were dispersed in 64 μL of ethanol and 64 μL of isopropanol and sonicated for 20 min, before addition of 16 µL of 5 *wt.*% binder solution and further sonication for 10 min. It should be noted that PVDF and PIL, are not alcohol soluble, so the binders were dissolved in DMF. After sonication, 16 µL of the ink were drop casted on the GC, resulting in a nominal total catalyst loading of 2 mg cm^-2^, and a nominal loading of 0.2 mg cm^-2^ palladium.

For the bulk electrolysis, the ground Pd-C powder was drop casted on carbon paper of a 2 cm^2^ area (1 cm^2^ per side). Before deposition, the carbon paper was first washed by sonication successively in acetone, ethanol and water, for 20 min each time, and eventually dried at 60 °C overnight. Then, an ink was prepared by sonicating 15 mg of catalyst and 2.5 mg PILs or PVDF (50 μL of 5 *wt.*%) binder in a total volume of 600 µL of DMF for 20 min. In the case of Nafion, since it was the only alcohol-soluble binder, the ink was prepared by 550 μL ethanol- isopropanol solution (50:50 *wt %*) and 50 μL of Nafion 5 *wt%* solution. For Fumion, 25 μL of 10 *wt%* solution was dispersed in DMF along with the Pd-C catalyst. 100 µL of the resulting ink were drop casted on each side of the carbon paper, resulting in an overall loading of ~2.5 mg cm^-2^.

- 1. Electrochemical studies

The electrochemical studies were performed using a Gamry Interface 1010 Potentiostat/ Galvanostat. All solutions were purged with N_2_ before the electrochemical studies. The preliminary voltammetric investigation for the hydrogenation of MA and MBY was conducted using a GC electrode (3 mm diameter, ALS), on top of which 5.8 uL of Pd-C/ Nafion were drop casted, again resulting in a nominal loading of 2 mg cm^‑2^.

For the Rotating Disk Electrode (RDE) experiments, a rotation rate control unit by Pine instruments was used. The rotation rate ranged from 0 to 2000 rpm and constant N_2_ bubbling was employed throughout the measurements. A Ag/AgCl electrode (saturated KCl) from ALS and a graphite rod were used as Reference and Counter Electrodes (RE, CE), respectively. Before each experiment, the graphite rod was cleaned by sonicating for 5 minutes in ethanol and 5 minutes in water.

For the bulk electrolyses, a divided cell was used, with a Nafion 117 membrane separating the anodic and cathodic compartments. The Nafion membrane was treated beforehand with 3 % v/v H_2_O_2_ for one hour, millipure H_2_O for one hour, 1 M HNO_3_ for two hours and finally with millipure H_2_O for one more hour, each step taking place at 80 °C, and the membranes were stored in 1 M H_2_SO_4_. Each cell compartment was filled with 23 mL of solution. All electrolyses were conducted potentiostatically for 2 hours, under constant catholyte stirring at 800 rpm and N_2_ bubbling. The cathodic compartment hosted the WE and RE (Ag/AgCl, saturated KCl), while a platinum wire CE was hosted in the anodic compartment. Dynamic current interrupt was employed to alleviate ohmic losses, unless otherwise stated. Electrochemical hydrogenation of benzaldehyde was conducted in 3 M acetate buffer, while the electrochemical hydrogenation of MA and MBY were carried out in 0.2 M H_2_SO_4_, and 0.1 M NaOH, respectively.

All potential values are reported with respect to the Reversible Hydrogen Electrode (RHE), using the following equation.

$$E_{RHE}=E_{Ag/AgCl}+0.197 V+0.059\times pH$$

The Faradaic Efficiency % (FE) of the electrochemical hydrogenations was calculated through the following equation, where *F* is the Faraday constant (96,485 C mol^-1^), C the final concentration in products, *V* the volume of solution, *n* the number of exchanged electrons (2 in all cases), and *Q_total_* the total charge that was passed.

$$FE \left( \% \right)= \frac{C \times V\times F\times n}{Q_{total}}$$

- 1. Products quantifications

The products of benzaldehyde hydrogenation were separated and detected using HPLC with a UV-Vis detector and chromatographs were recorded at 215 and 250 nm. A Thermo Fisher Hypersilic Gold C18 column was used and the elution was conducted isocratically, using a mobile phase of 57:43 H2O:Acetonitrile ratio and a flow rate of 1 mL min^-1^. External calibration curves were constructed using known ratios of benzaldehyde and benzyl alcohol for the quantification of reactants and products. For each sample, 100 uL solution aliquots where diluted in 900 uL of millipure water prior to injection.

For MA and MBY hydrogenations, all products were quantified through ^1^H NMR spectroscopy using an Agilent 400 MHz spectrometer after the dilution of 50 µL of the electrolysis solution in 500 µL of D_2_O containing 0.01 M of dioxane as internal standard.

In the case of MA ECH no species other than maleic acid and succinic acid were detected, so the yield calculations were conducted under the assumption the total concentration in organic acids (MA + SA) is constant. Similarly, for MBY ECH, we assume that the total concentration of MBY, 2-methyl-3-buten-2-ol (MBE, *semi-*hydrogenated product) and 2-methyl-3-butan-2-ol (MBA, fully hydrogenated product) is constant. We assumed that other than the organics’ reduction, only Hydrogen Evolution Reaction (HER) was occurring at the cathode.

The yield of succinic acid, as well as the yields and selectivities of MBE and MBA were determined according to its proportion in the aliquots. Note that routine voltammetric and EIS measurements run before bulk electrolysis led to a small product formation ~~(< 5 %)~~, which was taken into account and removed from the reported data.

$$SA_{yield} \left( \% \right)=100\times\frac{[SA]}{\left[ MA \right]+\left[ SA \right]}$$

$${MBE}_{yield} \left( \% \right)=100\times\frac{\left[ MBE \right]}{\left[ MBY \right]+\left[ MBE \right]+\left[ MBA \right]}$$

$${MBE}_{selectivity} \left( \% \right)=100\times\frac{[MBE]}{\left[ MBE \right]+[MBA]}$$

- 1. Transient OCP and pH measurements

For the *in-operando* measurements of transient Open Circuit Potential (OCP) changes and the corresponding local pH changes were recorded based on the protocol reported by Sauvé *et al.*^3^ The Pt-C/binder ink was prepared by dispersing 1.7 mg of Pt‑C (20 *wt %* Pt) powder in 140 uL solutions of ethanol-isopropanol mixtures (50-50 wt%.) containing 14.55 uL of 5%Nafion, or 12.1 uL of 5% PVDF or PIL solution. 20 uL of the dispersion were drop-casted on a 5 mm diameter GC RDE tip by Pine. The acetate buffer solution was saturated with H_2_, with constant bubbling throughout the measurements. The Pt-C/binder GC RDE electrode was polarized at current densities of 5, 10, 20 and 50 mA cm^-2^ for 3 minutes and then the OCP transients were measured for 5 minutes, with a time resolution of 30 ms, under a rotation rate of 400 rpm. All Pt-C/binder measurements were conducted using three different electrode deposits, to ensure result reproducibility.

1. **Supporting data**


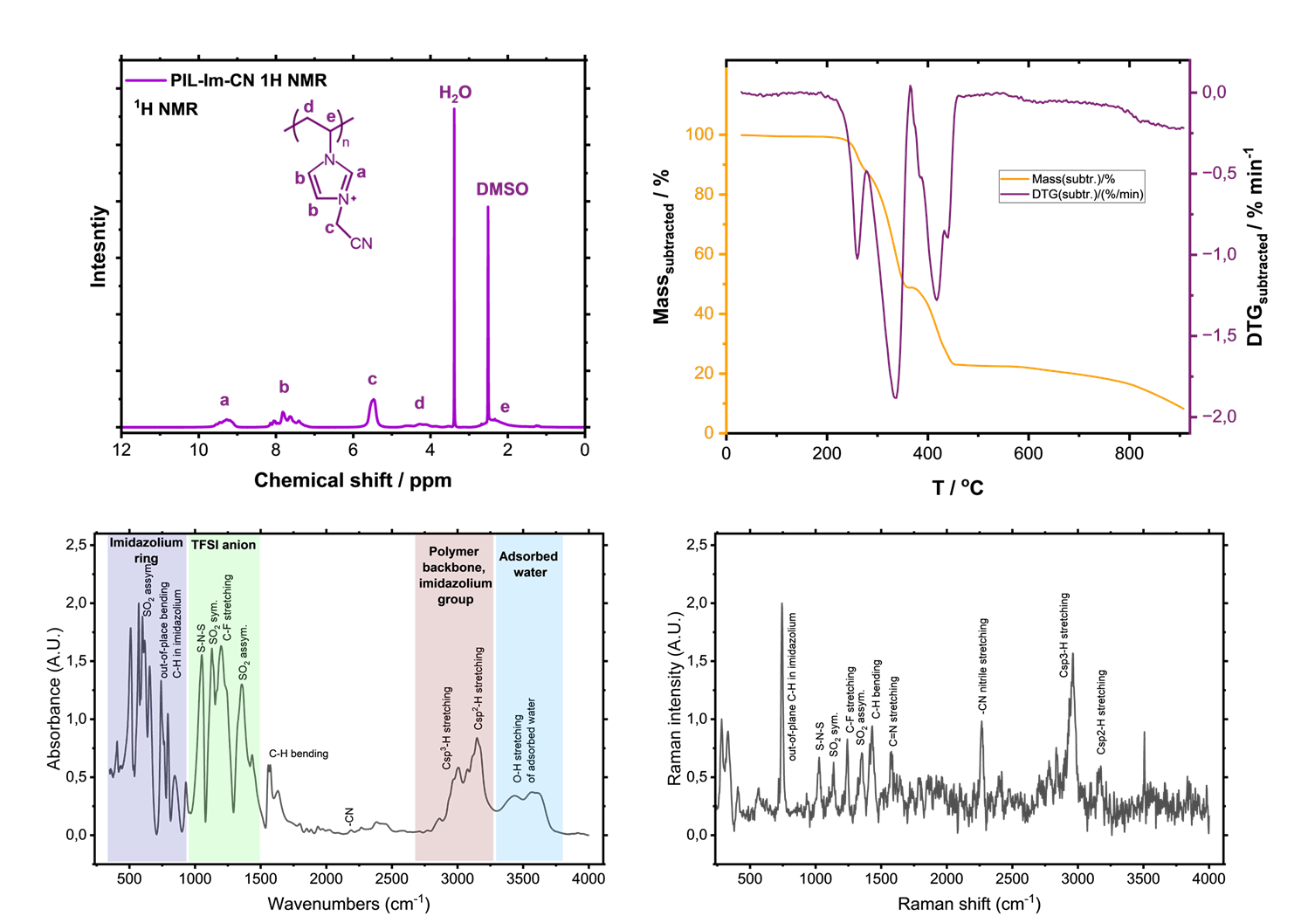


**Figure S1***: PIL-Im-CN characterization a) 1H-NMR spectrum in DMSO-d6; b) Thermogravimetric analysis; c) FTIR spectra and d) Raman spectra*

Both FTIR and Raman spectra confirm the structure of the PIL-Im-CN polymer. The spectra are composed of “blocks of peaks” corresponding to: (i) adsorbed water strongly interacting with imidazolium group (3250-3700 cm^-1^); (ii) polymer backbone and imidazolium cation (3200 – 1800 cm^-1^); (iii) TFSI anion (1500 – 1000 cm^-1^); imidazolium ring (950 – 500 cm^-1^).


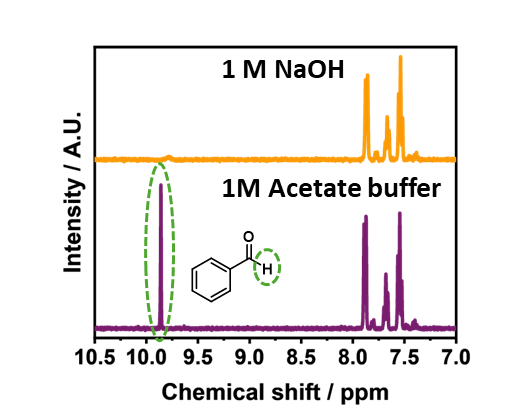


**Figure S2**: 1H NMR spectra of benzaldehyde in 1 M NaOH and 1 M acetate buffer

The acetate buffer was chosen as the optimal electrolyte for BZH ECH because for more acidic solutions the competing Hydrogen Evolution Reaction (HER) would be augmented, thus reducing the resulting FE values. Although alkaline electrolytes further suppress HER compared to acetate buffer, they also catalyze aldehyde side reactions, such as the gem diol formation (Reaction S1, Figure S3) and the Cannizzaro reaction, where benzaldehyde is disproportionated to benzyl alcohol and benzoic acid (Reaction S2, Figure S4).


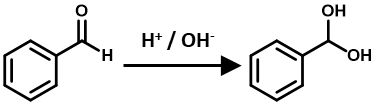


**Reaction S1**: Benzaldehyde gem-diol formation


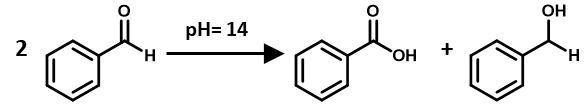


**Reaction S2**: Cannizzaro reaction of benzaldehyde

^1^H NMR spectra of BZH in 1 M NaOH electrolyte reveal that the carboxylic proton yields a miniscule signal, indicating that the redox-active carboxylic group is affected by the alkaline conditions (Figure S2).


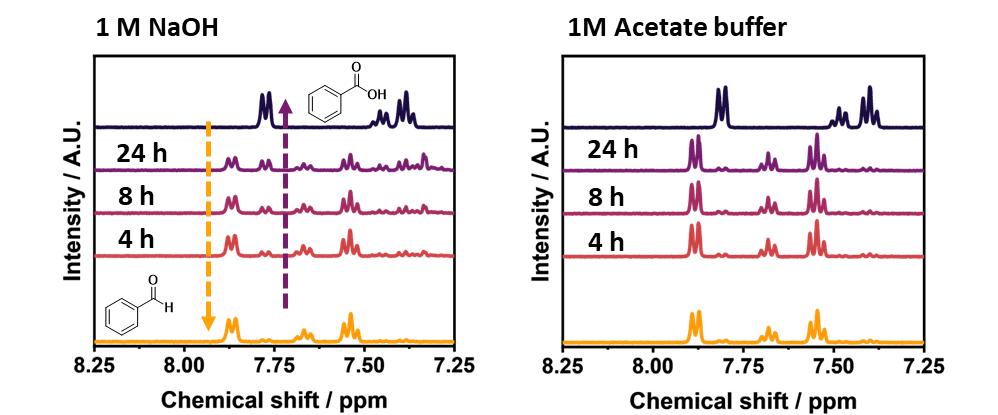


**Figure S3**: ^1^H NMR spectra to monitor the Cannizzaro reaction in 1 M NaOH and 1 M acetate buffer

In 1 M NaOH the reaction proceeds, where over time benzoic acid is formed (Figure S3). Contrastingly, the acetate buffer limits such side reactions, therefore ensuring the reactant’s (BZH) stability in the chosen electrolyte.


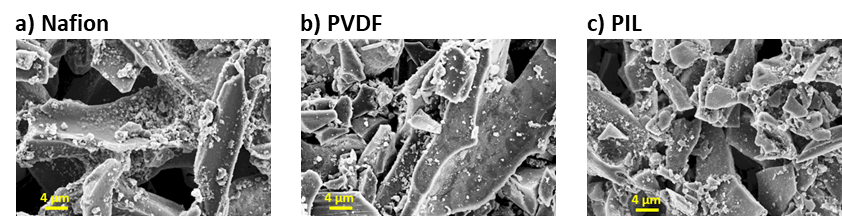


**Figure S4**: SEM images of the as prepared Pd-C/binder on carbon paper electrodes


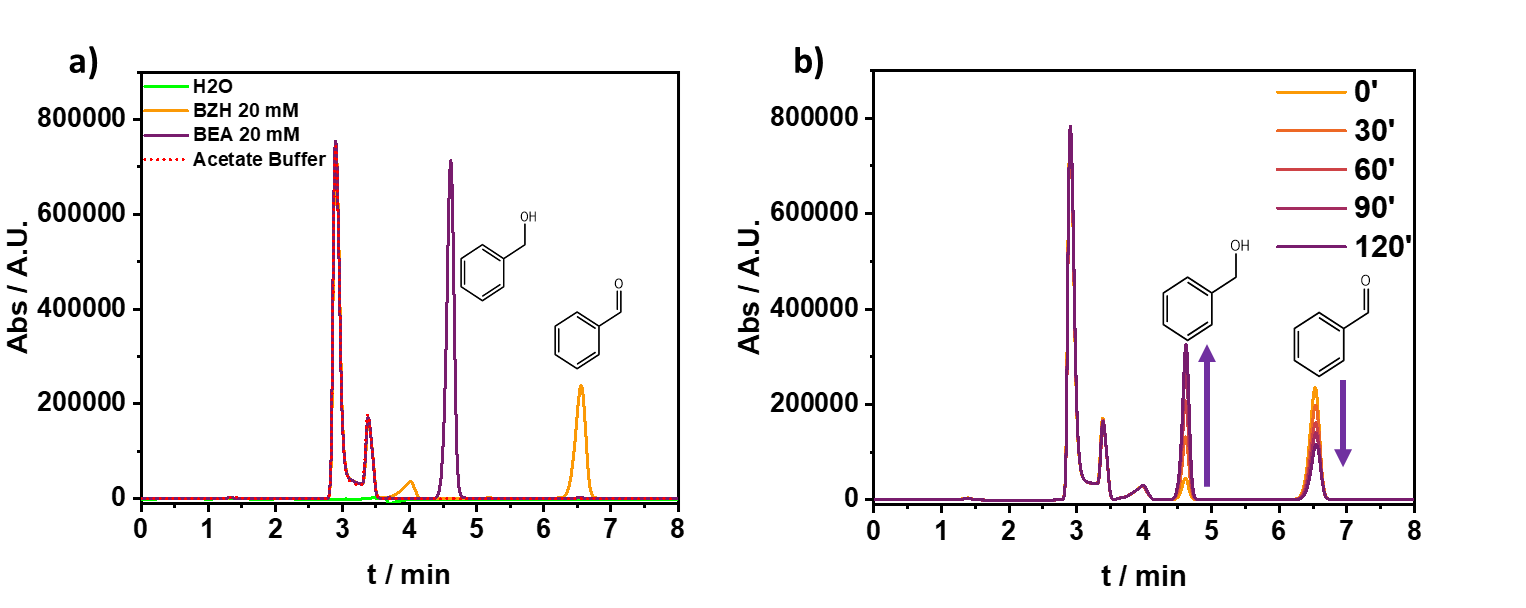


**Figure S5:** a) Chromatographs of milli-Q H_2_O, supporting electrolyte (acetate buffer, 3 M), 20 mM of benzaldehyde (BZH) and 20 mM of benzyl alcohol, at 215 nm. b) Chromatographs of the electrolysis solution over the course of the reaction for Pd-C/PIL electrode at -0.1 V vs. RHE, with dynamic current interrupt at 215 nm.

The chromatographs showed good separation between benzaldehyde and benzyl alcohol using UV-detection at 215 nm. It should be noted that two poorly separated peaks with elution times of 3 and 3.4 minutes stem from the acetate buffer supporting electrolyte, as they were present in all chromatographs that included the buffer solution, but not pure water injections. Additionally, one additional, ill-shaped peak was present for pure benzaldehyde, which can either correspond to benzoic acid, the oxidation product under ambient conditions, or other impurities. The intensity of this peak grew linearly with increasing benzaldehyde concentration (not shown here) and remained stable over the course of the electrolyses.


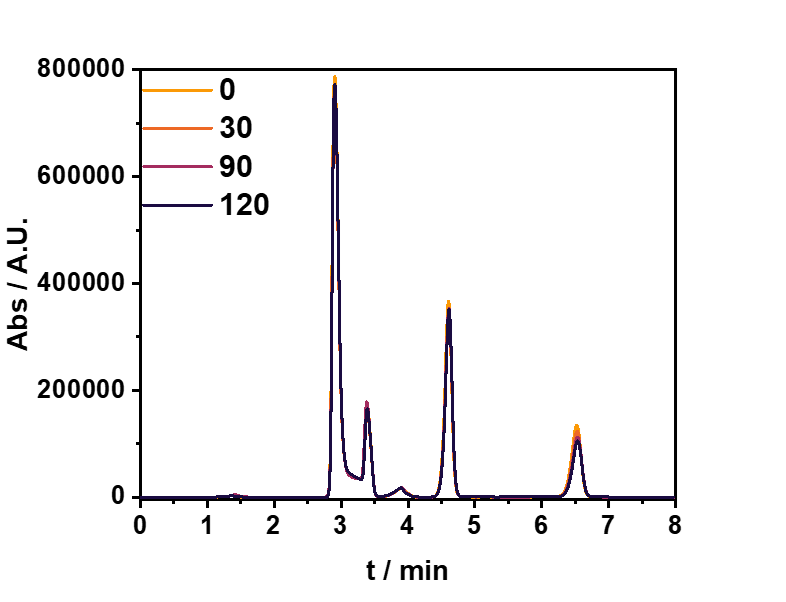


Slight loss of benzaldehyde was observed during the two-hour process due to its volatility (Figure S2), while benzyl alcohol showed no significant loss. Given that the calculated Faradaic efficiencies (FE) are impacted only by the moles of products and not reactants in the solution, the loss of benzaldehyde is not accounted for in the calculations.

**Figure S6**: **Blank test.** Electrolysis solution, containing 10 mM of benzaldehyde and 10 mM of benzyl alcohol in 3 M acetate buffer, over the course of 2 hours. No electrodes were present in the cell. The loss of benzaldehyde is due to its volatility, no spontaneous hydrogenation was observed.


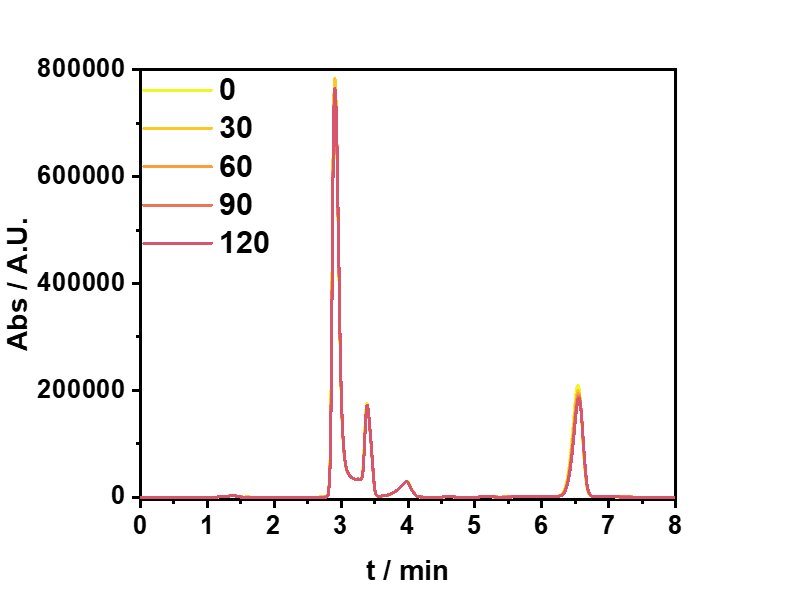


**Figure S7**: Chromatograph of electrolysis solution, over the course of 2 hours, using the plain carbon paper current collector at ‑0.1 V vs. RHE. No formation of benzyl alcohol was noted.


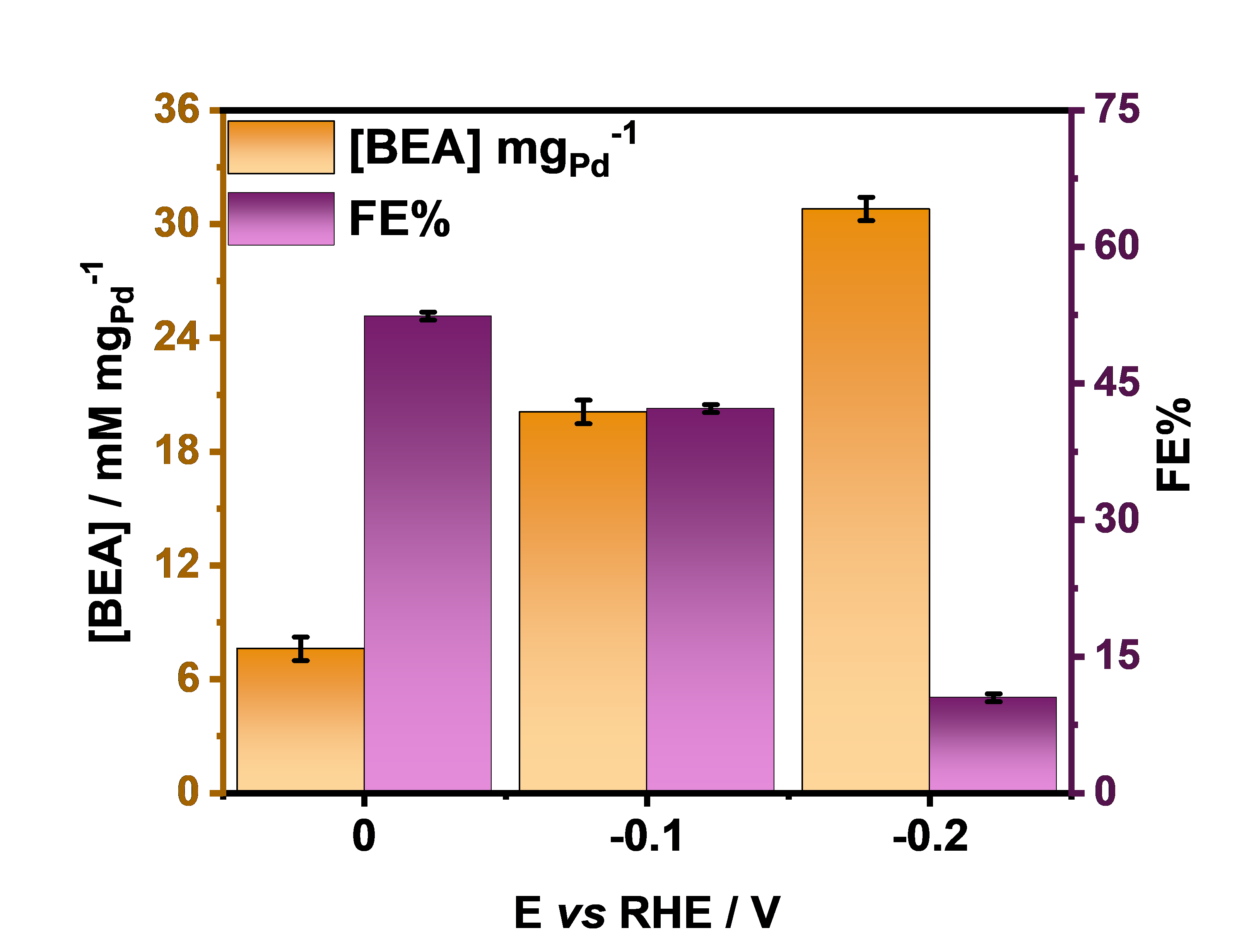


**Figure S8:** Benzyl alcohol formation and Faradaic efficiencies after 2 hours of electrolysis at different electrode potentials, using dynamic current interrupt, in 3 M acetate buffer, containing 20 mM of benzaldehyde, using Pd-C/PIL electrodes


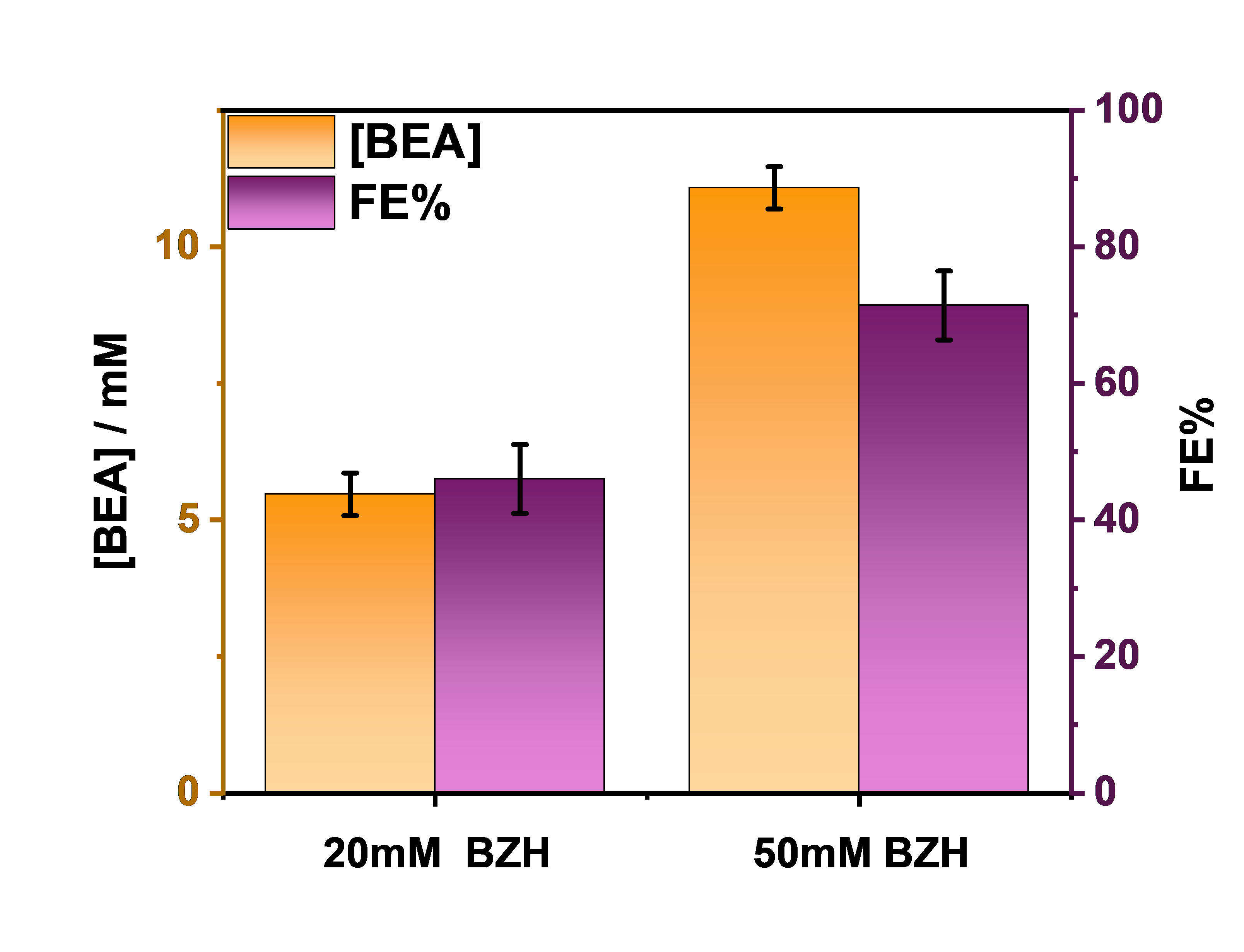


**Figure S9**: Benzyl alcohol formation (mM) and Faradaic efficiencies after 2 hours of electrolysis at -0.1 V vs. RHE, using dynamic current interrupt, in 3 M acetate buffer, containing 20 or 50 mM of benzaldehyde, using Pd-C/PIL electrodes


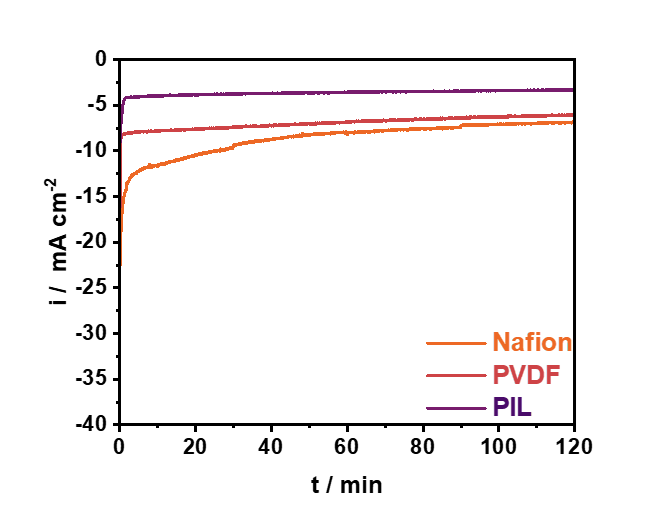

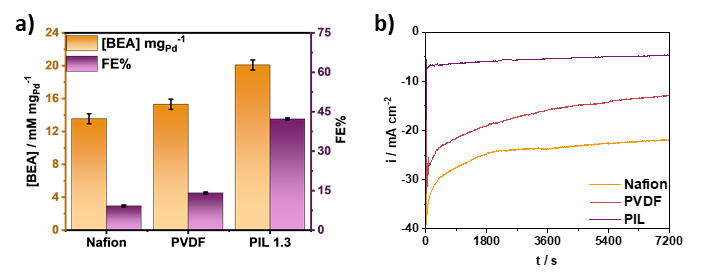


**Figure S10:** Chronoamperometry curves of Pd-C with different binders with no iR compensation. All curves recorded at -0.1 V vs. RHE for 2 hours, in 3 M acetate buffer, containing 20 mM benzaldehyde

**Figure S11**: Bulk electrolysis of BZH using dynamic current interrupt a) BEA formation and Faradaic efficiencies after 2 hours of electrolysis at -0.1 V vs. RHE, with iR compensation b) corresponding CA curves. All experiments conducted in 3 M acetate buffer, containing 20 mM of benzaldehyde, using Pd-C electrodes with different binders.


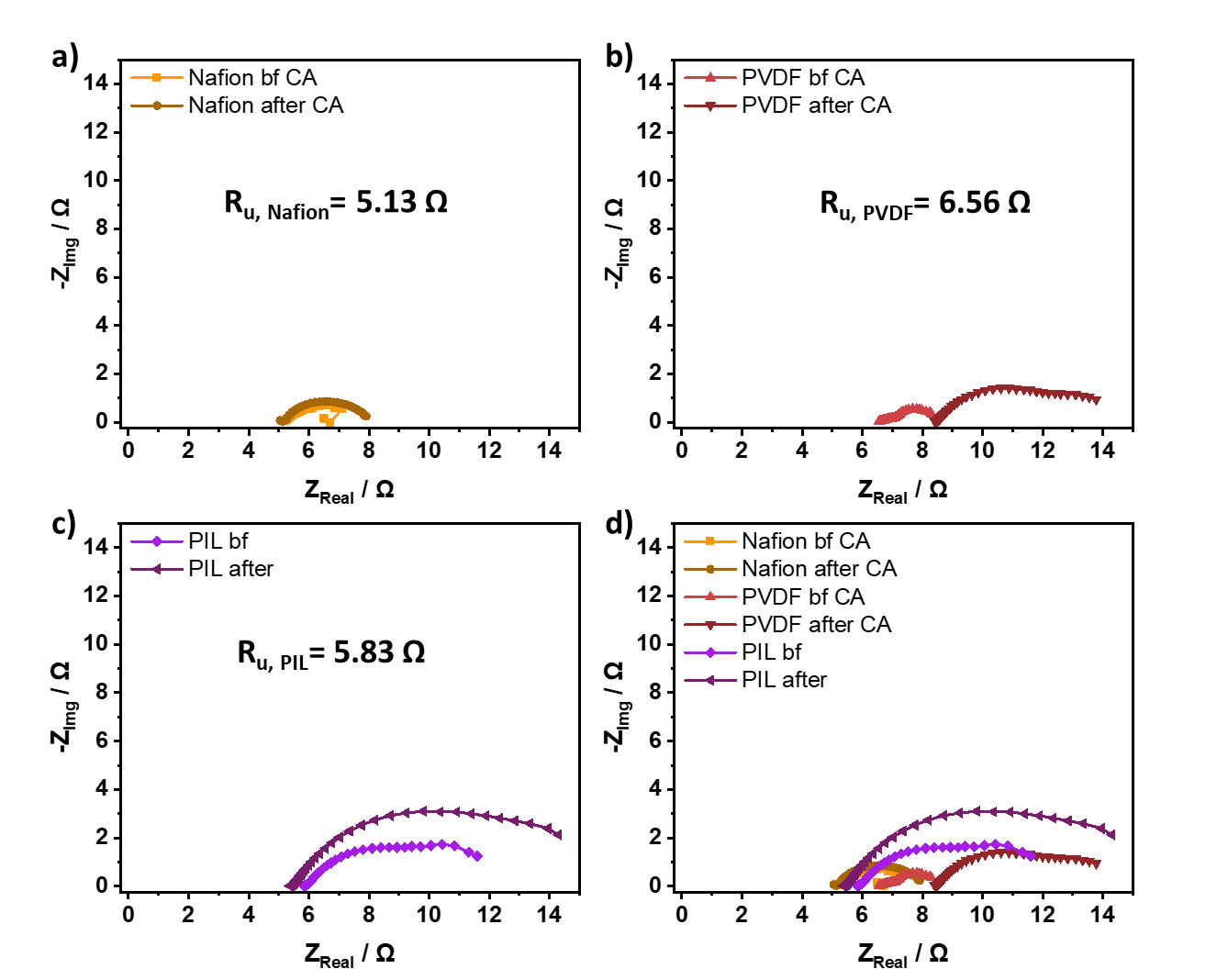


**Figure S12**: Potentiostatic Nyquist plots at -0.1 V vs. RHE, before and after 2 hours of electrolysis of 20 mM benzaldehyde in 3 M at -0.1 V, with dynamic current interrupt for a) Pd-C/ Nafion b) Pd-C/ PVDF, c) Pd-C/PIL electrodes d) all plots overlayed. R_u_ values correspond to the uncompensated resistance before the electrolyses.

As seen from the high frequency region of the Nyquist plots, the different Pd-C/binder electrodes exhibited similar uncompensated resistance (R_u_) values, indicating that neither the innate resistivity of the electrodes nor the presence of evolving hydrogen bubbles is the primary reason for the different performance of the electrodes. Different ohmic losses (IR-frop) have therefore to be attributed simply to different values of recorded current, I.

After 2 hours of electrolysis with iR compensation, the Pd-C/PIL and Pd-C/PVDF electrodes exhibited an increase in charge transfer resistance, potentially owing to the irreversible adsorption of organics on the Pd-C catalyst.


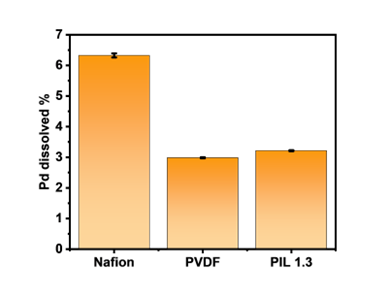


**Figure S13**: Palladium % dissolved, calculated from catalyst leaching in the electrolysis solution, after 2 hours of electrolysis at ­‑0.1 V vs. RHE, using dynamic current interrupt, in 3 M acetate buffer, containing 20 mM benzaldehyde, for different binders. Pd content in solution quantified through ICP-OES measurements. Error bars obtained by three independent ICP measurements.


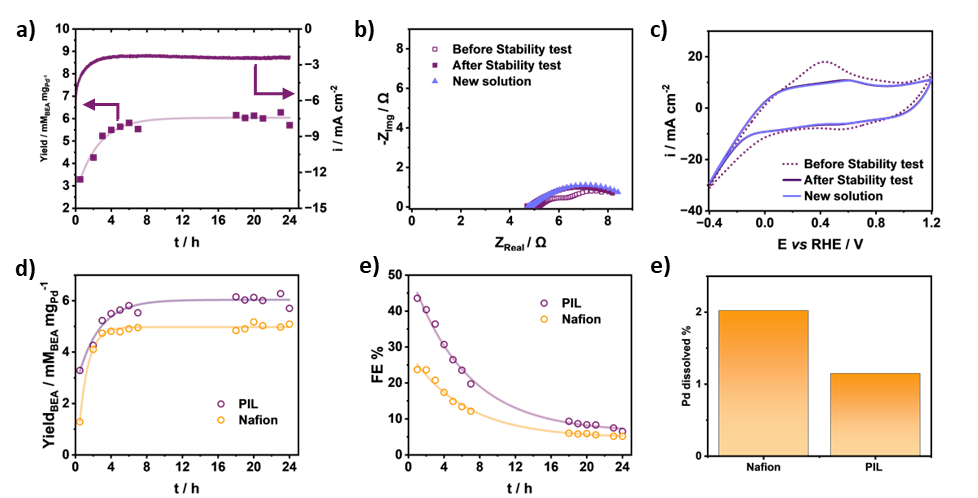


**Figure S14**: Pd-C/binder stability test a) BEA yield on Pd-C/PIL and corresponding CA curve b) Nyquist plots at -0.1 V before and after the stability test, also with a fresh BZH solution c) cyclic voltammograms before and after the stability tests, also with a fresh solution d) BEA yield comparison of Pd-C/PIL and Nafion electrodes and e) corresponding Faradaic efficiencies e) amount of Pd dissolved from Pd-C electrode with Nafion and PIL. All stability tests were carried out for 24 hours at -0.1 V vs RHE, without current interrupt, in an upscaled H-Cell


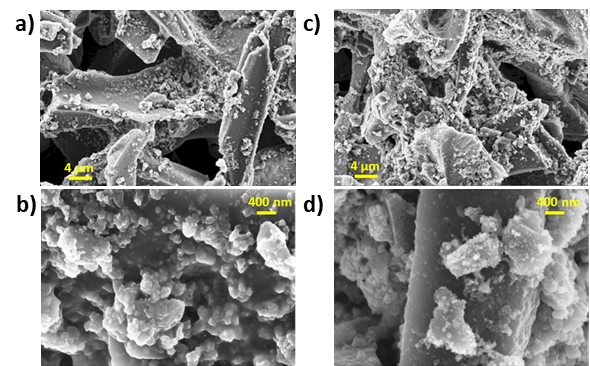


**Figure S15**: SEM images of Pd-C/Nafion on carbon paper, a,b before and c,d after the 24-hour stability test


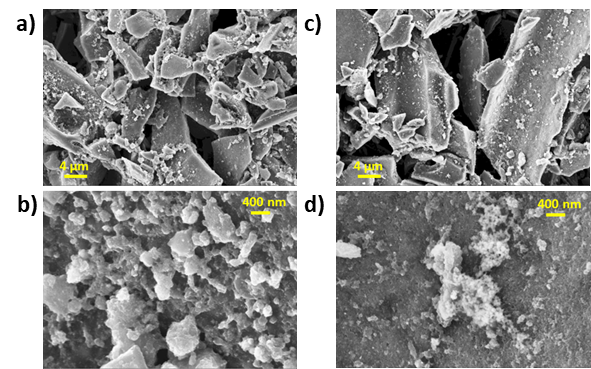


**Figure S16**: SEM images of Pd-C/PIL on carbon paper a,b before and c,d after the 24-hour stability test


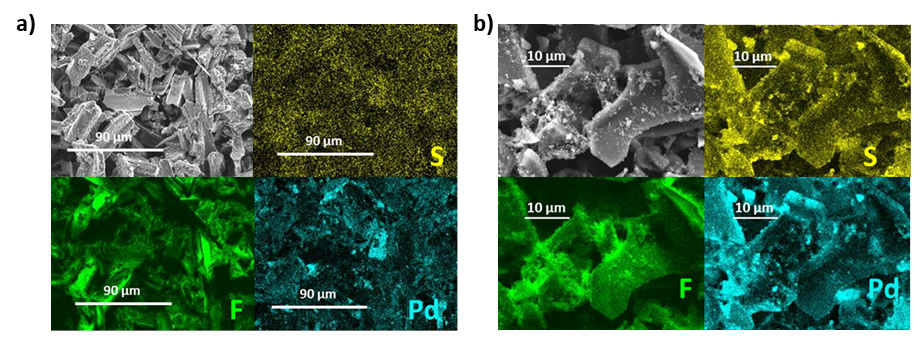


**Figure S17**: EDX mapping of Pd-C/Nafion a) before b) after the 24-hour stability test


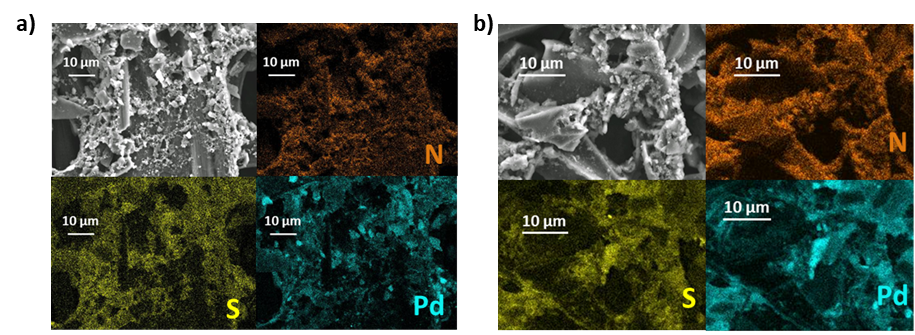


**Figure S18**: EDX mapping of Pd-C/PIL a) before b) after the 24-hour stability test


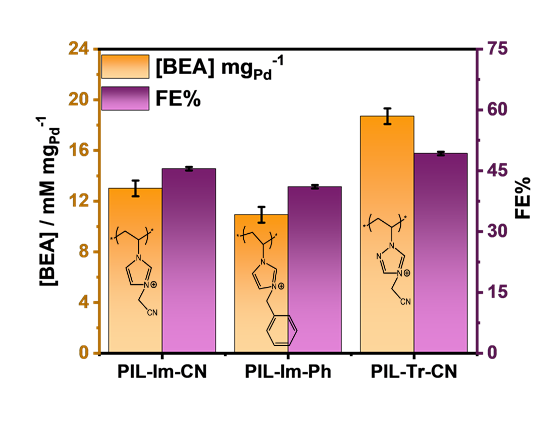


**Figure S19**: Benzyl alcohol formation and Faradaic efficiencies after 2 hours of electrolysis at -0.1 V vs. RHE, with no iR compensation, in 3 M acetate buffer, containing 20 mM of benzaldehyde, using Pd-C electrodes with PIL binders with different chemical structures. It should be noted that PIL-Im-Ph has a Br^-^ anion, in contrast to PIL-Im-CN and PIL-Tr-Cn (TFSI^-^ anions), making it a fluorine free binder.


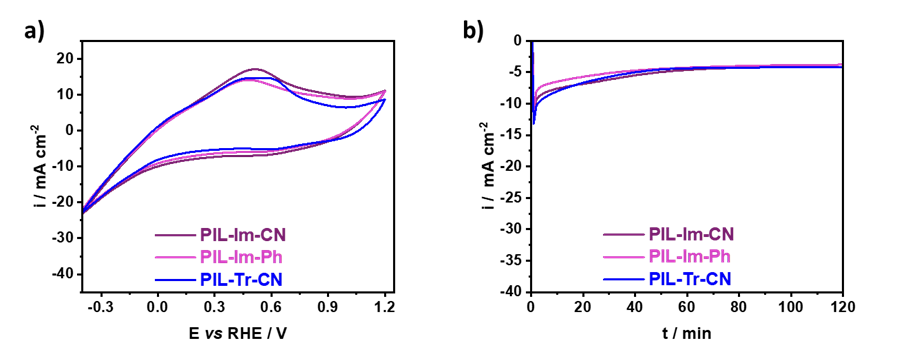


**Figure S20**: Electrochemical performance of Pd-C with different PIL structures a) CVs of Pd-C/ PIL on CP, in 3 M acetate buffer with 20 mM BZH, 50 mV s^-1^ b) Chronoamperometric curves during the electrolysis of 20 mM BZH at -0.1 V, no iR compensation

The initial PIL‑Im‑CN used in the preceding experiments was compared to a synthesized PIL-Im-Ph (in which the cyanomethyl group is replaced by a benzyl group, and the TFSI^-^ with a Br^-^ anion) and PIL-Tr-CN (in which the imidazolium ring is replaced by a 1,2,4-triazonium ring, while retaining the cyanomethyl group; Figure S11). Preliminary CVs showed no significant changes in the electrochemical behavior of Pd-C with the change of the PILs’ chemical structure (Figure S12a). Additionally, the current densities during the electrolysis of BZH were comparable (Figure S12b). However, bulk electrolysis under identical conditions showed that PIL-Tr-CN exhibited the highest yields, while PIL-Im-Ph exhibiting only slightly lower FE and yield, likely due to its more hydrophobic side group. Thus, it is shown that by tuning the PIL’s chemical structure, even better ECH yields can be achieved, potentially with F-free PILs as well.

*
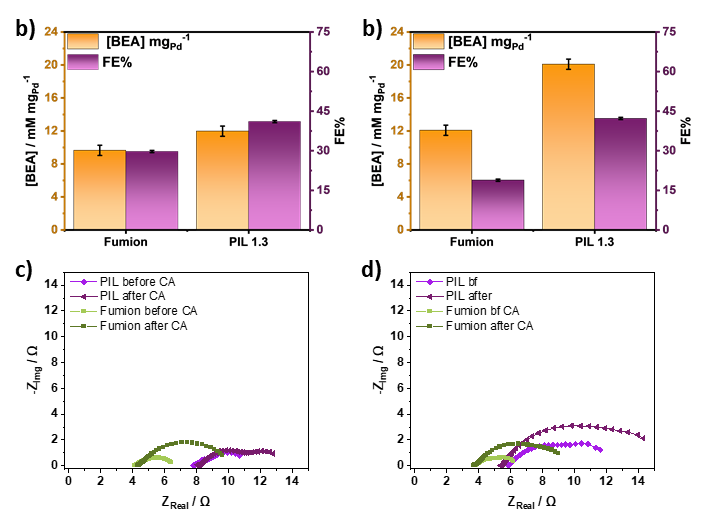
*

**Figure S21**: Comparison between Pd-C/ Fumion and Pd-C/ PIL for BZH ECH. a) BEAformation and Faradaic efficiencies after 2 hours of electrolysis at -0.1 V vs. RHE, with no iR compensation b) same with iR compensation c) EIS measurements at -0.1 V vs RHE, before and after electrolysis without iR compensation d) EIS measurements at -0.1 V vs. RHE before and after electrolysis with iR compensation. All measurements were conducted in 3 M acetate buffer, containing 20 mM of benzaldehyde, using Pd-C electrodes


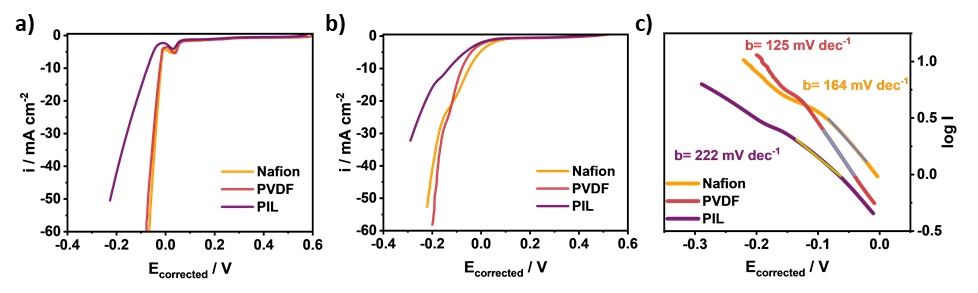


**Figure S22**: iR-corrected LSVs and respective Tafel plots for Pd-C electrodes a) iR-corrected LSVs in the absence of BZH b) iR-corrected LSVs with 20 mM BZH c) respective Tafel plots. LSVs recorded in 3 M Acetate buffer, on a RDE, 1600 rpm, 5 mV s^‑1^, shown in Figures 3a,c in the manuscript, Tafel plots of S13a shown in Figure 3b.

For the voltammetric experiments, we exclude the possibility of significantly different exchange current densities, since the electroactive material (Pd) is the same for all electrodes. In the absence of BZH, each binder shows two different Tafel slopes, showing different kinetic regimes. At potential relevant to bulk electrolysis, the slopes for Nafion and PVDF are very close to the Volmer-step limiting process (~120 mV dec^-1^), while PIL’s 166 mV dec^-1^ indicates a mixed-controlled region (Figure S13b). The increased slopes in the presence of BZH, signify that the kinetics of electrochemical reactions becomes more complex, with probably the chemical step become rate-limiting. Similar Tafel slope increase in the presence of organics has been reported elsewhere.^4^


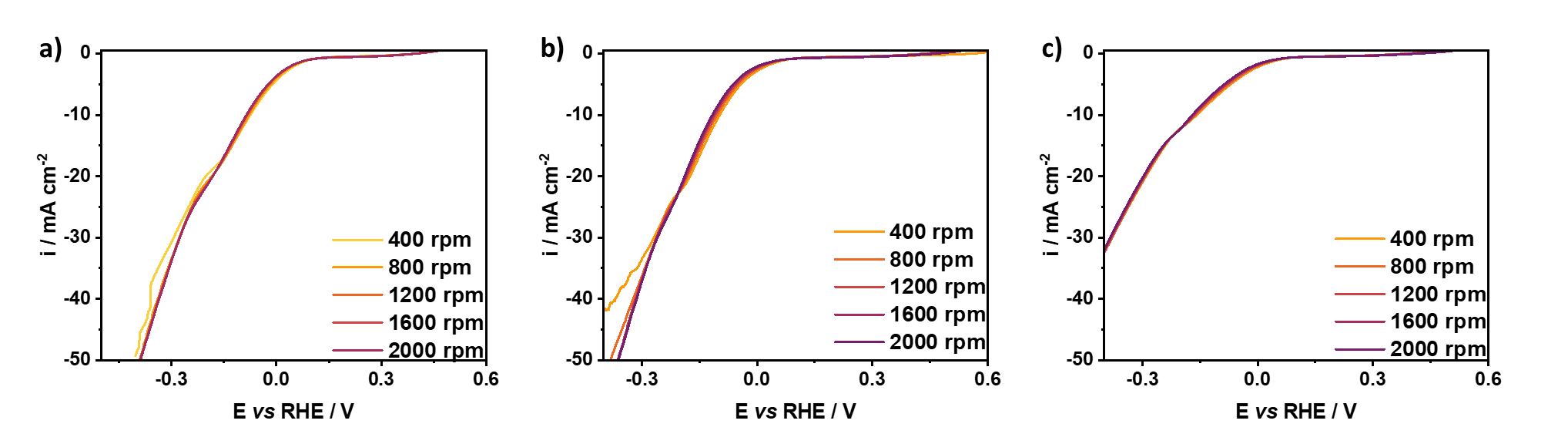


**Figure S23**: LSVs in 3 M acetate buffer containing 20 mM benzaldehyde, with 5 mV s^-1^, for different rotation rates, for a) Pd-C/ Nafion b) Pd-C/ PVDF c) Pd-C/PIL


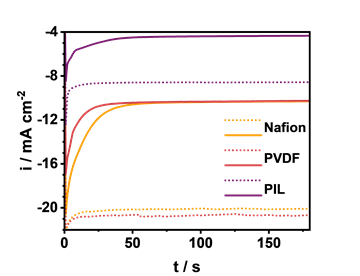


**Figure S24**: Chronoamperometry measurements for Pd-C/binder on GC RDE at -0.1 V, 1600 rpm. Dotted lines correspond to the absence of benzaldehyde, solid lines correspond to 20 mM benzaldehyde

**Figure S25:** XPS spectra of 3d Pd, from the Pd-C/binder inks using 3 different binders.

We assessed the impact of binders on the electronic properties of Pd NPs by analyzing Pd 3d XPS spectra of the deposited inks (Figure S1**)**. Each spectrum shows two sharp peaks at 337.8 and 343.2 eV, corresponding to the Pd 3d₅/₂ and Pd 3d₃/₂ spin-orbit components, respectively. Notably, PVDF and PIL exhibit shoulders at lower binding energies, indicating chemical interactions and partial charge transfer between these binders and Pd, with PVDF causing a more pronounced effect.

*
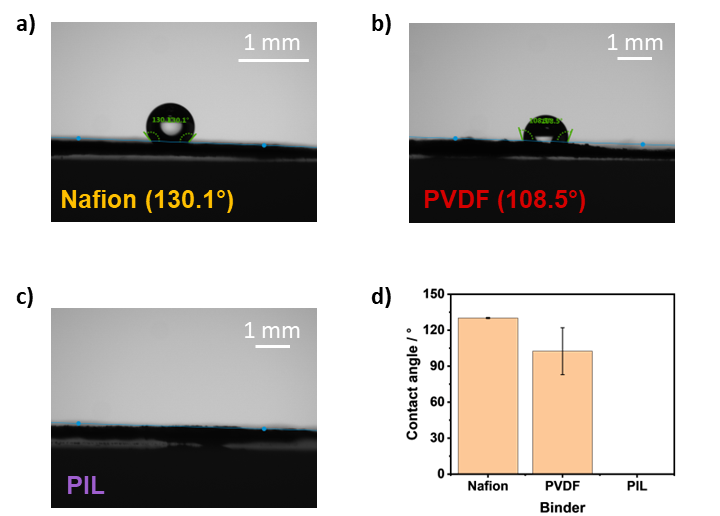
*

**Figure S26:** Contact angle measurements of Pd-C/binder on carbon paper with a) Nafion b) PVDF c) PIL d) contact angle values

Prior to the pH swing measurements, the Pt-C/PIL electrode and the Pt ring’s OCP values were monitored, to ensure that the electrode’s potential values indicative of Pt HER/HOR equilibrium potential.


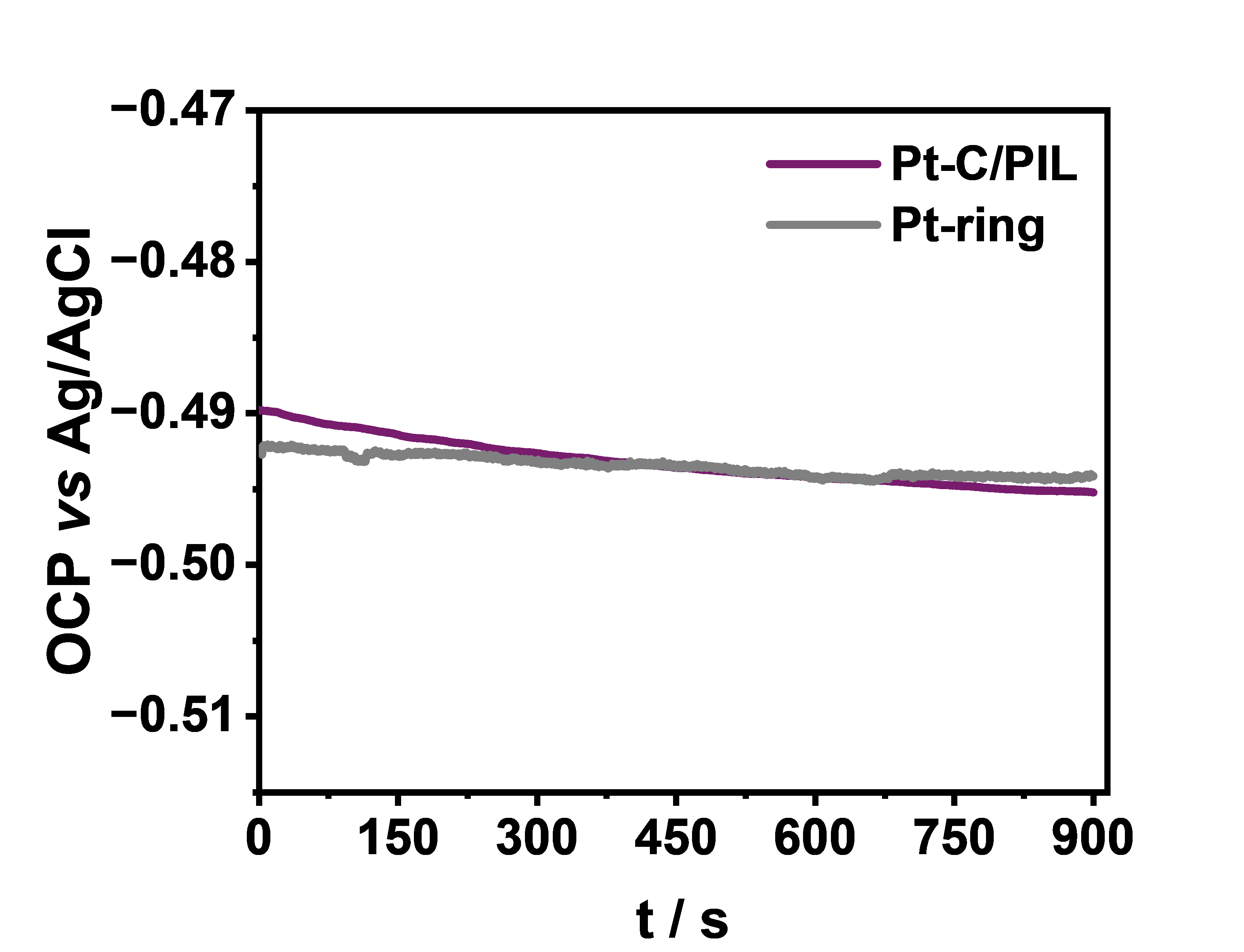


**Figure S27:** OCP comparison of Pt ring and Pt-C/PIL in H_2_ saturated acetate buffer for pH swing measurements


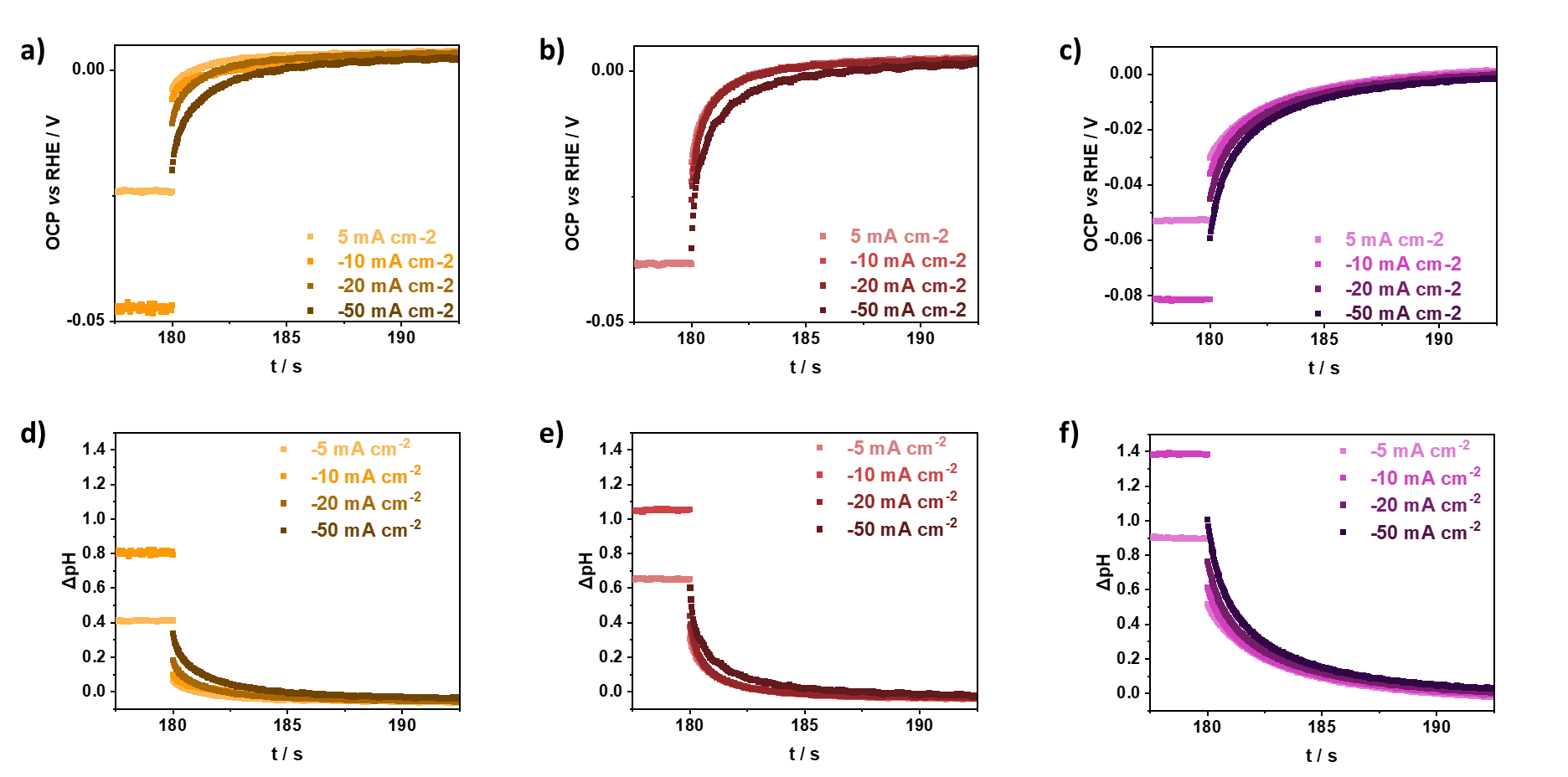


**Figure S28**: OCP and pH responses of Pt-C/ binder electrodes on GC RDE after polarization at different current densities in H_2_– saturated 3 M acetate buffer, 400 rpm: OCP curves after polarization a) Pt-C/ Nafion on GC RDE b) Pt-C/ PVDF on GC RDE c) Pt-C/ PIL on GC RDE and d-f their respective pH changes


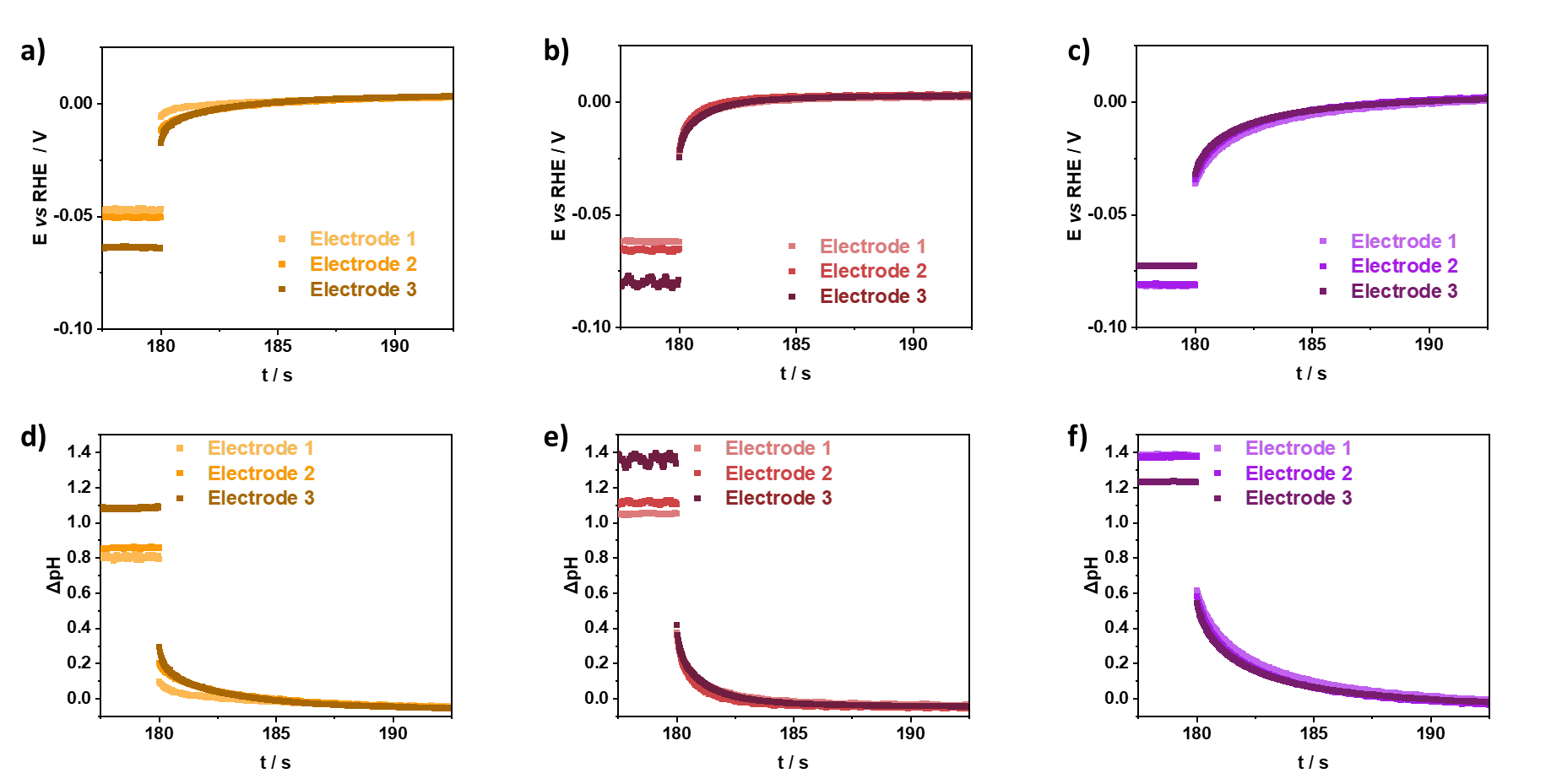


**Figure S29**: OCP and pH responses of Pt-C/ binder electrodes after polarization at -10 mA cm^-2^ for 3 different electrodes on GC/RDE in H_2_– saturated 3 M acetate buffer, 400 rpm: OCP curves after polarization a) Pt-C/ Nafion on GC RDE b) Pt-C/ PVDF on GC RDE c) Pt-C/ PIL on GC RDE and d-f their respective pH changes

**
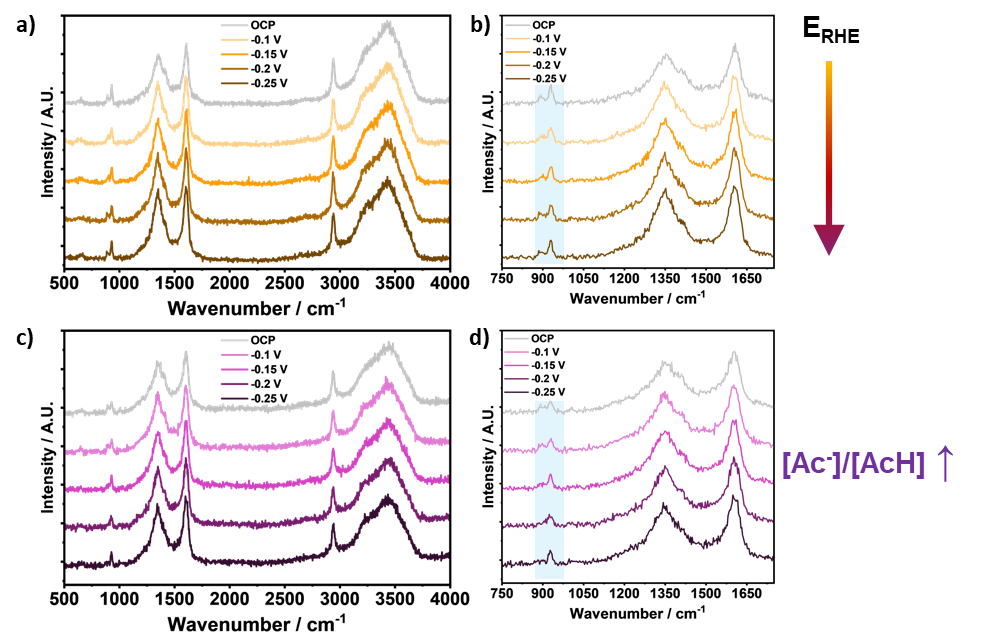
**

**Figure S30**: In-situ Raman spectra on Pd-C/binder under different applied potential with a) Nafion^®^, c) PIL in acetate buffer. Plots b,d correspond to the zoomed in spectra at the acetate/acetic acid region (shaded region).

**Figure S31**: logarithm of current density vs. overpotential for Pt-C/ binder on GC RDE electrodes in H_2_-saturated 3 M acetate buffer, 400 rpm

**Figure S32**: CVs of Pd-C/ Nafion in 0.2 M H_2_SO_4_ in the absence and presence of 0.1 M MA, 50 mV s^-1^

.

**Figure S33**: Cyclic Voltammograms of Pd-C/Nafion in 0.1 M NaOH, in the absence and presence of 0.1 M MBY, 50 mV s^-1^

**Table S1**: Pd-C/binder loadings for bulk electrolyses. Final loadings (in mg) of Pd-C/binder on carbon paper (1 cm^2^ per side, 2 cm^2^ in total). The loadings indicate that in spite of the lower loading, Pd-C/PIL exhibited comparable if not better ECH performance.

| **Experiment** | **Pd-C/Nafion** | **Pd-C/PVDF** | **Pd-C/PIL** |
| --- | --- | --- | --- |
| BZH ECH, no CI | 6.22 | 5.92 | 5.3 |
| BZH ECH | 5.85 | 5.97 | 4.58 |
| MA ECH | 5.6 | 4.97 | 3.22 |
| MBY ECH | 6.24 | 5.58 | 5.55 |

**Table S2**: BEA generation and FE% using 3 different Pd-C/PIL-Im-Ph electrodes. Electrolyses were conducted at -0.1 V vs. RHE, with no iR correction, for 2 hours in 3 M Acetate buffer.

| Electrode no. | Pd-C/binder  loading / mg | R_u_ / Ω | [Benzyl Alcohol] / mM | [Benzyl Alcohol] / mM mg_Pd_^-1^ | FE % |
| --- | --- | --- | --- | --- | --- |
| #1 | 5.58 | 6.5 | 5.01 | 10.54 | 41.47 |
| #2 | 5.9 | 7.75 | 4.72 | 9.31 | 41.05 |
| #3 | 5.73 | 3.13 | 4.90 | 10.01 | 40.60 |
| **Average** | **5.74** | **5.79** | **4.88** | **9.95** | **41.04** |
| **Standard Deviation** | **0.16** | **2.39** | **0.15** | **0.62** | **0.44** |
| **RSD %** | **2.79** | **41.25** | **3** | **6.2** | **1.06** |

**Supplementary note 1: Mechanism of BZH ECH**

The electrochemical hydrogenation of benzaldehyde has been thoroughly studied on Pd-C electrodes, yet there are different interpretations of its mechanism.^4,6–9^ In accordance with preexisting literature, we observed the suppression of voltammetric features related to hydrogen adsorption/ absorption and its reoxidation. *Quasi*-steady state LSVs verified this, as seen in **Figures 3a,c**, where the Pd-H formation peak disappeared and another reduction shoulder appeared, corresponding to the hydrogenation of benzaldehyde.

Hydrogenation reactions can occur electrochemically either through a Proton Coupled Electron Transfer (PCET) or through the reaction between the electrochemically adsorbed hydrogen and the adsorbed organic species (Electrochemical Hydrogenation, ECH).^10^ LSVs recorded for different rotation rates overlap almost completely for the entire potential range studied, as seen in **Figure S23**. This denotes that the reaction always occurs under pure kinetic control (either electrochemical or/and chemical) with no proton or organic species mass transport limitations. This points to the reaction of adsorbed organics with electrochemically adsorbed hydrogen (H_ads_), following a Langmuir-Hinshelwood mechanism.^11^ Given that the reactants have to adsorb before they react and the number of active sites is fixed on a given electrode, enhancing mass transfer by increased rotation rates, does not yield higher reaction rates, and consequently, faradaic currents. Had the reaction been diffusion limited with the reactants in solution reacting at the vicinity of the electrode, the reaction rate would be augmented by increasing the mass transfer coefficient (k_m_ ∝ ω^1/2^), thus we would observe increasing currents with increasing rotation rates.

ECH and HER are competing reactions, given that benzaldehyde adsorption reduces the coverage of electrosorbed hydrogen that further reacts/recombines to produce H_2_ according to HER mechanism,^4,12,13^ while they are at the same time interrelated as they both require the presence of electrosorbed H. All this means that best ECH catalytic electrodes should result in the optimum θ_Η_ coverage value.

HER starts occurring at higher rates in the absence of organics, as seen in the chronoamperometric measurements in **Figure S24**, but when benzaldehyde is added, HER is hindered by the presence of organics due to competitive adsorption. On the other hand, ECH rate increases with overpotential, as indicated by the increase in benzyl alcohol yield from the bulk electrolyses for 0 to -0.2 V *vs.* RHE (**Figure S8**). However, as the reductive overpotential increases further, the potential-dependent θ_Hads_ increases, until it reaches a value where adsorbed hydrogen obstructs the adsorption of organics, thus limiting their subsequent hydrogenation and HER is the main reaction occuring.^8^ This dynamic relationship between the two reactions also explains the decrease in Faradaic efficiency when the applied potential reaches -0.2 V *vs.* RHE. Finally, the competition between benzaldehyde ECH and HER was verified by using increased amounts of benzaldehyde (50 mM) and achieving both higher yields and higher FEs (**Figure S9**).

Apart from the competition for active sites, the irreversible adsorption of organics (either benzaldehyde or the electrochemically formed benzyl alcohol) must be considered. In this case, the organics form an inhibiting adlayer on the catalyst’s surface.^4^ This speculation is in line with the increase in R_ct_ as seen in the Nyquist plots of the different electrodes after the electrolysis of benzaldehyde (**Figure S12**), were the increase of R_ct_ can indicate the strong adsorption of organics on the catalyst’s surface, hindering charge transfer.

In summary, benzaldehyde hydrogenation goes through a surface mediated reaction. The ECH reaction is in direct competition with HER due to competing adsorption, with ECH suppressing HER at low overpotentials, and *vice versa* when θ_Hads_ increases with increasing overpotential. This dynamic relationship has, in turn, a direct impact on the faradaic efficiency of ECH, which is hampered in case HER dominates.

**Supplementary note 2: Qualitative correlation of pH and Volmer step rate**

The impact of local pH can be qualitatively described using equations S1-S3. As it can be seen, the Volmer step current (∝ Volmer step rate) decreases with increasing pH, as also experimentally observed by Surendranath et al.^5^ We refrain from further extracting the exact values of reaction rate, as this model qualitatively correlates the aforementioned quantities.

The Volmer step dependence on pH can be formulated as follows:

| $i_{V}=F*\left[ H^{+} \right]*k^{0}{exp}^{\frac{-0.5*F*\eta}{R*T}}$ | Eq. S1 |
| --- | --- |
| $[H^{+}]={10}^{-pH}$ | Eq. S2 |
| $i_{V}=F*{10}^{-pH}*k^{0}{exp}^{\frac{-0.5*F*\eta}{R*T}}$ | Eq. S3 |

*Where i_V_ is the Volmer step current, η is the applied overpotential, k^0^ is the HER kinetic constant, F (=96485 C mol^‑1^) is Faraday’s constant, and assuming for a cathodic transfer coefficient a_c_=0.5*

**References**

1. Zhao, Q., Zhang, P., Antonietti, M. & Yuan, J. Poly(ionic liquid) Complex with Spontaneous Micro-/Mesoporosity: Template-Free Synthesis and Application as Catalyst Support. *J. Am. Chem. Soc.* **134**, 11852–11855 (2012).

2. Yuan, J., Giordano, C. & Antonietti, M. Ionic Liquid Monomers and Polymers as Precursors of Highly Conductive, Mesoporous, Graphitic Carbon Nanostructures. *Chem. Mater.* **22**, 5003–5012 (2010).

3. Sauvé, E. R., Tang, B. Y., Razdan, N. K., Toh, W. L., Weng, S. & Surendranath, Y. Open circuit potential decay transients quantify interfacial pH swings during high current density hydrogen electrocatalysis. *Joule* **8**, 728–745 (2024).

4. Sanyal, U., Yuk, S. F., Koh, K., Lee, M., Stoerzinger, K., Zhang, D., Meyer, L. C., Lopez‐Ruiz, J. A., Karkamkar, A., Holladay, J. D., Camaioni, D. M., Nguyen, M., Glezakou, V., Rousseau, R., Gutiérrez, O. Y. & Lercher, J. A. Hydrogen Bonding Enhances the Electrochemical Hydrogenation of Benzaldehyde in the Aqueous Phase. *Angewandte Chemie* **133**, 294–300 (2021).

5. Tang, B. Y., Bisbey, R. P., Lodaya, K. M., Toh, W. L. & Surendranath, Y. Reaction environment impacts charge transfer but not chemical reaction steps in hydrogen evolution catalysis. *Nat Catal* **6**, 339–350 (2023).

6. Song, Y., Sanyal, U., Pangotra, D., Holladay, J. D., Camaioni, D. M., Gutiérrez, O. Y. & Lercher, J. A. Hydrogenation of benzaldehyde via electrocatalysis and thermal catalysis on carbon-supported metals. *Journal of Catalysis* **359**, 68–75 (2018).

7. Birkett, M. D. & Kuhn, A. T. The electrochemical reduction of Benzaldehyde. *Electrochimica Acta* **25**, 273–278 (1980).

8. Meyer, L. C., Sanyal, U., Stoerzinger, K. A., Koh, K., Fulton, J. L., Camaioni, D. M., Gutiérrez, O. Y. & Lercher, J. A. Influence of the Molecular Structure on the Electrocatalytic Hydrogenation of Carbonyl Groups and H _2_ Evolution on Pd. *ACS Catal.* **12**, 11910–11917 (2022).

9. Yang, Q., Ge, B., Yuan, P., Luo, S., Zhang, H., Zhao, Z., Zhang, J., Wang, S., Bao, X. & Yao, X. Amine Coordinated Electron-Rich Palladium Nanoparticles for Electrochemical Hydrogenation of Benzaldehyde. *Advanced Functional Materials* **33**, 2214588 (2023).

10. Zhang, P. & Sun, L. Electrocatalytic Hydrogenation and Oxidation in Aqueous Conditions ^†^. *Chin. J. Chem.* **38**, 996–1004 (2020).

11. Bondue, C. J. & Koper, M. T. M. A mechanistic investigation on the electrocatalytic reduction of aliphatic ketones at platinum. *Journal of Catalysis* **369**, 302–311 (2019).

12. Singh, N., Sanyal, U., Fulton, J. L., Gutiérrez, O. Y., Lercher, J. A. & Campbell, C. T. Quantifying Adsorption of Organic Molecules on Platinum in Aqueous Phase by Hydrogen Site Blocking and in Situ X-ray Absorption Spectroscopy. *ACS Catal.* **9**, 6869–6881 (2019).

13. Sasaki, K., Kunai, A., Harada, J. & Nakabori, S. Electrolytic hydrogenation of phenols in aqueous acid solutions. *Electrochimica Acta* **28**, 671–674 (1983).
